# Genetic association of lipids characteristics and lipid lowering drug target genes with sepsis

**DOI:** 10.1371/journal.pone.0331023

**Published:** 2025-09-11

**Authors:** Yu Wang, Haiyue Zhang, Yuanyuan Zhan, Zhuoran Li, Sujing Li, Yingchao Zhang, Shubin Guo

**Affiliations:** 1 Emergency Medicine Clinical Research Center, Beijing Chao-Yang Hospital, Capital Medical University, Beijing, P.R. China; 2 Thrombosis Research Center, Beijing Jishuitan Hospital, Capital Medical University, Xicheng District, Beijing, China; 3 Department of Endocrinology, Beijing Chao-Yang Hospital, Capital Medical University, Beijing, P.R. China; 4 Department of Functional Examination, National Clinical Research Center for Cancer/Cancer Hospital, National Cancer Center, Chinese Academy of Medical Sciences, Peking Union Medical College, Beijing, China; 5 Department of dermatology, Zhengzhou People’s Hospital, Zhengzhou, China; University Hospital Zurich: UniversitatsSpital Zurich, SWITZERLAND

## Abstract

**Background:**

Sepsis is a severe systemic infection that can result in organ dysfunction and mortality. Dyslipidemia emerges as a key player in the intricate web of sepsis pathogenesis. Yet, the causal relationship between blood lipid profiles and sepsis risk remains uncertain. This study aims to investigate the association between genetically predicted lipid traits, drug targets, and sepsis.

**Methods:**

The UK Biobank’s Genome-wide association studies (GWAS) produced data on lipid and apolipoprotein characteristics. Four independent GWAS datasets were used to generate the sepsis statistics. The study utilized the two-sample Mendelian randomization (MR) approach, which incorporates multivariable (MVMR) models, to assess the correlations between sepsis risk and lipid-related parameters. To gain further insight, expression quantitative trait loci (eQTL) data were used to investigate the significant drug targets for lipid-lowering.

**Results:**

Increasing ApoA-1 levels was associated with a diminished risk of sepsis (under 75) (OR 0.927, 95% CI 0.861–0.999; p = 0.047). This inverse correlation persevered even after performing multivariable MR. Elevated levels of HDL-C were associated with a decreased risk of sepsis (under 75) (OR 0.897, 95% CI 0.838–0.960; P = 0.002) and incidence of sepsis (OR 0.883, 95% CI 0.820–0.951; P = 0.001), which was consistent across sensitivity analyses. Furthermore, a decrease in total cholesterol exhibited a causal effect on sepsis in multivariable MR (OR 0.779, 95% CI 0.642–0.944; P = 0.01). The genetic variants related to lowering LDL-C, located near the HMGCR and LDLR genes, were predicted to elevate the risk of sepsis. Moreover, genetic mimicry near the ANGPTL3 and LPL gene suggested that reducing the activity of ANGPTL3 and LPL (mimicking antisense anti-ANGPTL3 and LPL agents) was forecasted to decrease sepsis risk.

**Conclusion:**

Genetically inferred elevated ApoA-1, total cholesterol, and HDL-C manifest a protective effect against sepsis. Within the 9 lipid-lowering drug targets investigated ANGPTL3 and LPL exhibit potential as candidate drug targets for sepsis.

## Introduction

Sepsis, a major challenge for clinicians, is a deadly syndrome that ensues when the body’s immune system overreacts to an infection. The advent of septic shock and subsequent failure of multiple organs pose a serious risk to the patient’s life, particularly in severe cases [1,2]. Despite extensive study, sepsis still presents significant obstacles to effective treatment and early identification [3]. To improve patient outcomes, there is still an urgent need to promptly identify risk factors and devise novel therapeutic strategies.

Recent research has unveiled the association between hyperlipidemia, especially in critical illness situations, and a significantly amplified susceptibility to sepsis [4]. Several investigations have illuminated the markedly heightened risk of cardiovascular events that exists in sepsis and hyperlipidemia. Lipid irregularities may also serve as a pivotal link connecting vascular diseases to sepsis. Furthermore, studies by Elisabeth et al. and Matthew et al. affirm that the utilization of statins, a pharmacological class aimed at reducing cholesterol levels, in the management of hyperlipidemia, can improve endothelial function and reduce the risk of sepsis [5,6]. The causal connection between dyslipidemia and sepsis risk remains unknown. Nonetheless, evidence from observational studies may be biased by survival, reverse causality, and residual confounding.

Mendelian Randomization (MR) analysis, an approach that allows for the assessment of causal relationships and the identification of potential therapeutic targets, serves as an effective alternative to randomized clinical trials [7,8]. In this study, a two-sample MR was employed to examine the association between genetically predicted lipid parameters and the risk of sepsis. The univariate MR analysis demonstrated the effect between lipid features and sepsis risk, both directly and through interactions with other exposures. On the other hand, the multivariable MR analyses aimed to estimate the direct and independent causal effect of each exposure on an outcome. Moreover, MR can also use genetic variations that mimic the pharmacological inhibition of pharmacogenetic targets as instrumental variables. Through regression analysis, we endeavor to investigate the causal inference of the potential impact between the risk of sepsis and genetically predicted lipid modification at various gene targets.

Henceforth, this study endeavors to elucidate the causal relationship of lipids and apolipoproteins in the development of sepsis. We further conducted drug-targeted MR to explore the potential effects of lipid-lowering drug targets on the risk of sepsis.

## Methods

### Study design

**Fig 1:**
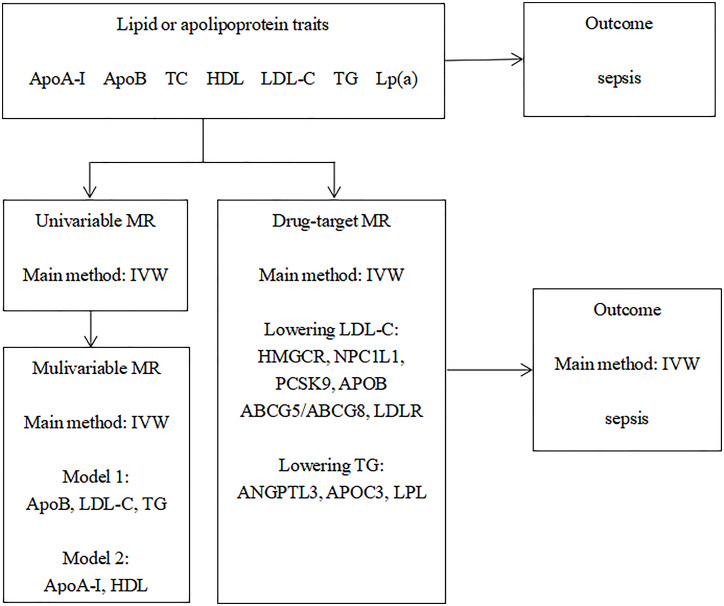
Overall study design. GWAS, genome-wide association study; Apo, apolipoprotein; HDL-C, high-density lipoprotein cholesterol; LDL-C, low-density lipoprotein cholesterol; TG, triglycerides; TC, total cholesterol; Lp(a), lipoprotein A; IVW, inverse-variance weighted.

This study followed the Strengthening the Reporting of Observational Studies in Epidemiology-Mendelian Randomisation reporting guidelines [9], while the study design is presented in Fig 1. To conduct this investigation, publicly accessible GWAS datasets and eQTL datasets were employed, for which prior informed consent and ethical approval had been acquired. Our study did not need ethical approval given that our investigation used publicly available summary statistics.

### GWAS data source

UK Biobank, an extensive cohort of adult volunteers in the United Kingdom, supplied summary statistical information regarding lipids and sepsis [10]. We selected four cohort studies as primary outcomes: The first cohort was the incidence of sepsis in the whole cohort of patients (total sepsis), the second cohort was the incidence of sepsis in patients under 75 years old (sepsis under 75), the third cohort was the incidence of sepsis requiring critical care (sepsis with critical care), and the forth cohort was the 28-day mortality in critical care admission (sepsis in 28-day mortality). The research entailed a sample size of 474841 control patients and 11643 cases of sepsis. Apolipoprotein A1 (Apo-A1), Apolipoprotein B (Apo-B), total cholesterol (TC), low-density lipoprotein cholesterol (LDL-C), triglyceride (TG), lipoprotein A (Lp(a)), and high-density lipoprotein cholesterol (HDL-C) were chosen as independent genetic variants meeting the criteria of a physical distance of 10,000 kb and an r^2 < 0.001 linkage disequilibrium (LD) clumping thresholds [11]. SNPs lacking sufficient proxies and missing data were excluded from this study.

### Genetic variant selection

For univariable MR analyses, we initially identified independent single nucleotide polymorphisms (SNPs), surpassing the linkage disequilibrium clumping r 2 threshold of 0.001 and with a window size of 10 Mb, that exhibited genome-wide significant (p < 5 × 10^−8^) in relation to each respective trait (Table 1, Supplement table 1 in S2 File). In the context of multivariable MR analyses, Model 1 encompassed all genome-wide significant SNPs associated with any of the traits including ApoB, LDL-C, TG, TC, and Lp(a). The characteristics in Model 2 were ApoA1 and HDL-C. Subsequently, we systematically eliminated the selected SNPs from the list if they demonstrated a significant association (p < 5 × 10−8) with the confounding factor of serious infection, which was verified on the PenoScanner website: www.phenoscanner.medschl.cam.ac.uk.

**Table 1:** Instrumental Variables details of lipids.

exposure	SNP	other_allele	effect_allele	eaf	exposure	SNP	other_allele	effect_allele	eaf	exposure	SNP	other_allele	effect_allele	eaf
ApoA-I	rs4784709	T	A	0.96	ApoB	rs8112973	A	G	0.98	HDL-C	rs4899251	C	T	0.94
	rs3732356	G	T	0.93		rs1229984	T	C	0.97		rs3732356	G	T	0.93
	rs9987289	A	G	0.91		rs4687614	G	A	0.95		rs9987289	A	G	0.91
	rs1661052	G	A	0.91		rs7590687	T	C	0.92		rs10786114	C	T	0.87
	rs7116797	A	G	0.89		rs2058122	T	C	0.86		rs686030	C	A	0.86
	rs10917383	A	G	0.88		rs473224	T	G	0.85		rs13144151	A	G	0.85
	rs686030	C	A	0.86		rs1277762	C	T	0.84		rs8086351	C	G	0.82
	rs1086056	T	G	0.84		rs9894946	A	G	0.84		rs3820897	T	C	0.82
	rs8086351	C	G	0.82		rs581080	G	C	0.82		rs10774439	G	A	0.81
	rs10023962	T	G	0.82		rs1556562	G	T	0.79		rs2159607	G	T	0.81
	rs7134035	C	T	0.82		rs278981	T	C	0.76		rs3825669	A	G	0.80
	rs13402475	C	G	0.82		rs10832963	T	G	0.74		rs2726114	A	G	0.80
	rs13326165	A	G	0.80		rs1250258	C	T	0.74		rs4239651	T	C	0.79
	rs4239651	T	C	0.79		rs6667939	C	T	0.72		rs1270076	A	G	0.78
	rs6467595	C	T	0.77		rs12471768	T	C	0.70		rs4947121	T	C	0.77
	rs1400362	T	C	0.74		rs11065384	T	C	0.69		rs9608783	A	G	0.77
	rs4660586	C	T	0.74		rs2784259	A	T	0.67		rs1993878	C	A	0.75
	rs4762756	T	C	0.74		rs969075	T	C	0.66		rs643186	T	C	0.75
	rs2804894	G	A	0.73	LDL-C	rs1229984	T	C	0.97		rs1509560	T	G	0.75
	rs140584594	A	G	0.73		rs204469	A	G	0.96		rs13111599	A	G	0.74
	rs2792751	T	C	0.73		rs9987289	A	G	0.91		rs7281183	G	A	0.74
	rs4795386	A	G	0.72		rs9894946	A	G	0.84		rs2804894	G	A	0.73
	rs2544654	G	T	0.72		rs7241918	G	T	0.82		rs2196808	T	C	0.73
	rs10750766	C	A	0.71		rs1556562	G	T	0.79		rs140584594	A	G	0.73
	rs681869	C	T	0.70		rs261334	G	C	0.79		rs1431659	A	G	0.73
	rs2642438	A	G	0.70		rs3732359	G	A	0.78		rs2792751	T	C	0.73
	rs2925979	T	C	0.70		rs10832963	T	G	0.74		rs10750766	C	A	0.71
TC	rs9987289	A	G	0.92		rs1250258	C	T	0.74		rs554146	A	C	0.70
	rs6662286	T	C	0.91		rs140584594	A	G	0.73		rs2642438	A	G	0.70
	rs2886232	T	C	0.88		rs2250802	G	A	0.72		rs2925979	T	C	0.70
	rs488490	C	A	0.87		rs6667939	C	T	0.72		rs2339234	G	A	0.68
	rs2156552	A	T	0.82		rs12471768	T	C	0.70		rs3027167	C	T	0.68
	rs581080	G	C	0.82		rs2642438	A	G	0.70					
LP(a)	rs2457569	C	G	0.11										
	rs9355291	C	T	0.06										
	rs7412	C	T	0.12										
TG	rs2305746	A	G	0.93		rs278981	T	C	0.76		rs1292065	C	G	0.71
	rs2811964	A	G	0.91		rs2773469	A	G	0.73		rs2925979	T	C	0.70
	rs1938566	C	T	0.83		rs9908820	A	G	0.73		rs326222	T	C	0.70
	rs3820897	T	C	0.82		rs11185542	G	C	0.73		rs6532798	C	T	0.70
	rs581080	G	C	0.82		rs2487294	G	T	0.72		rs7861679	C	T	0.70
	rs6800707	C	G	0.81		rs7704653	A	G	0.72		rs6792725	A	G	0.69
	rs9480889	C	G	0.78		rs7786102	G	A	0.72		rs7947951	A	G	0.69
	rs9902027	C	T	0.77		rs2131311	A	G	0.71		rs320369	A	G	0.68
	rs684773	A	C	0.77		rs729761	T	G	0.71		rs1420384	G	T	0.67
	rs10775406	A	G	0.76		rs10750766	C	A	0.71		rs28577186	G	A	0.66

SNP, single nucleotide polymorphisms; eaf, expected average frequency.

The SNPs shown in the table all met the instrumental variable filtering criteria shown in the Methods section. P-values for SNPs shown in the table are all less than 5E-8. More detailed information can be found in the Supplementary Table in S2 File.

A selection of commonly used lipid-lowering drugs and innovative therapeutics was made based on recent guidelines for the management of dyslipidemia. These medications include statins, ezetimibe, PCSK9 inhibitors, bile acid sequestrants, mipomersen, fibrates, angiopoietin-like 3 (ANGPTL3) inhibitors, and antisense oligonucleotide targeting apolipoprotein C-III (APOC3) mRNA [12,13]. The identification of genes encoding the pharmacological targets of these drugs was identified using the DrugBank database (https://go.drugbank.com/) and relevant reviews [14–16]. These target genes were further classified into two categories based on their primary pharmacological action: LDL-C-lowering target genes (LDL Receptor (LDLR), HMG-CoA reductase (HMGCR), Niemann-Pick C1-like protein 1 (NPC1L1), PCSK9, Apolipoprotein B-100 (APOB), ABCG5 and ABCG8) and TG-lowering target genes (lipoprotein lipase (LPL), PPARA, ANGPTL3 and APOC3). As no genetic variants of PPARA were found during the variant selection process, it was excluded from further evaluation. Considering the proximity of the genes encoding ABCG5 and ABCG8, variants in the vicinity of these genes were combined in our analyses. In conclusion, nine drug targets were included in the study: HMGCR, NPC1L1, PCSK9, APOB, ABCG5/ABCG8, LDLR, ANGPTL3, APOC3 and LPL. The instrumental variables were used to select SNPs that reduce protein activity, whicg are located within±100kb of gene loci that are associated with LDL-C or TG levels. In order to avoid the impact of strong linkage disequilibrium (LD) on the results, the threshold of LD was set (r2 < 0.3).

### Statistical analyses

After instrument harmonization and selection, we utilized the inverse variance-weighted (IVW) approach as the primary method for conducting our MR analysis [17]. To assess the potential presence of horizontal pleiotropy among instrumental variables, we employed MR-Egger regression and MR-PRESSO method [18,19]. Moreover, We also used Cochran’s Q statistic and MR–Egger test (intercept) to scrutinize heterogeneity and pleiotropy [18]. In cases where pleiotropy was observed, we employed the weighted median method as the preferred approach. To further investigate highly heterogeneous SNPs, we conducted a series of sensitivity analyses in our univariable MR.

Next, we proceeded with multivariable MR analysis employing the multivariable IVW method as our primary approach. SNPs exhibiting linkage disequilibrium with a threshold of r2 ≥ 0.001 were excluded from further analysis. Employing this approach, We assessed all the instrumental variables for lipid traits to discern their independent effects on sepsis.

Finally, the correlation between relevant lipid traits and each SNP at or near the drug target was ascertained utilizing the GWAS data obtained from UK Biobank. The IVW method was employed to determine the effect of each drug target on sepsis. The MR Egger regression equation was utilized to assess the horizontal pleiotropy of the genetic tool, with a p-value exceeding 0.05 providing evidence against the presence of horizontal pleiotropy [20].

All the aforementioned statistical analyses were performed utilizing the “TwoSample MR” (version 0.5.7) packages in the statistical program R (version 4.3.1) [21]. The criterion for statistical significance was established as p < 0.05.

## Results

In the MR, the F-statistics for all these SNPs exceeded 10 (Supplement Table 1 in S2 File). These results suggested that there is no potential weak instrument bias.

### Association of sepsis with lipid levels

Instrumental heterogeneity was observed at the statistical threshold of 0.05 when analyzing the causal effect of HDL-C on sepsis, ApoA-I on sepsis (sepsis under 75), and TC on sepsis (sepsis with critical care) (Cochran’s Q test, p < 0.05; Supplement Table 2 in S2 File); Consequently, we used the random-effects IVW method. Conversely, no evidence of instrumental heterogeneity was found (Cochran’s Q test, p > 0.05); thus leading us to employ the fixed-effects IVW method.

**Fig 2:**
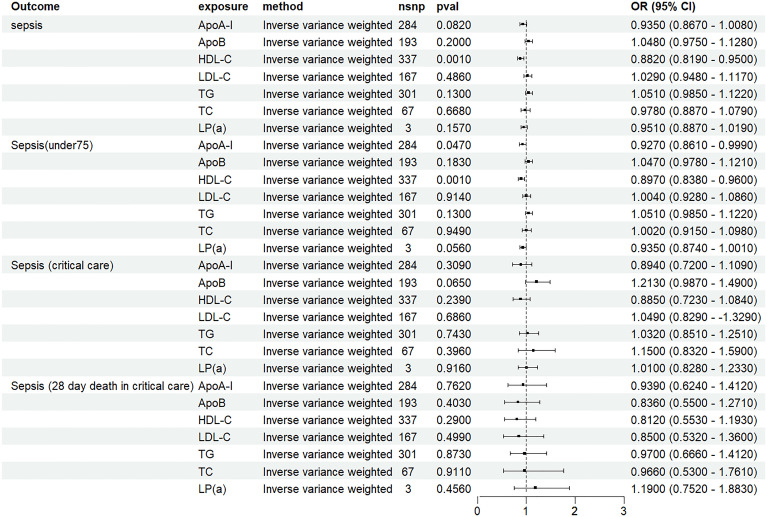
Univariable Mendelian randomization results. N SNP, number of single nucleotide polymorphisms; OR, odds ratio; CI, confidence interval; Apo, apolipoprotein; HDL-C, high-density lipoprotein cholesterol; LDL-C, low-density lipoprotein cholesterol; TG, triglycerides; TC, total cholesterol; Lp(a), lipoprotein A.

In the univariable MR analysis, we discovered an association between ApoA-I and sepsis (under75) (OR 0.927, 95% CI 0.861–0.999; p = 0.047). Furthermore, we observed that HDL was associated with sepsis (OR 0.883, 95%CI 0.820–0.951; P = 0.001) and sepsis (under 75) (OR 0.897, 95%CI 0.838–0.960; P = 0.002), respectively (Fig 2, Supplement Table 2 in S2 File). Based on the Inverse variance weighted, MR – Egger, weighted median, weighted mode, and simple mode method, the effect estimate remained consistent with the IVW estimate (Supplement Figures 1-7 in S1 File). We did not detect any evidence of horizontal pleiotropy (Supplement Table 3 in S2 File). Subsequently, the robustness of the findings was confirmed through the leave-one-out sensitivity analysis, as illustrated in Supplement Figures 8-14 in S2 File.

**Fig 3:**
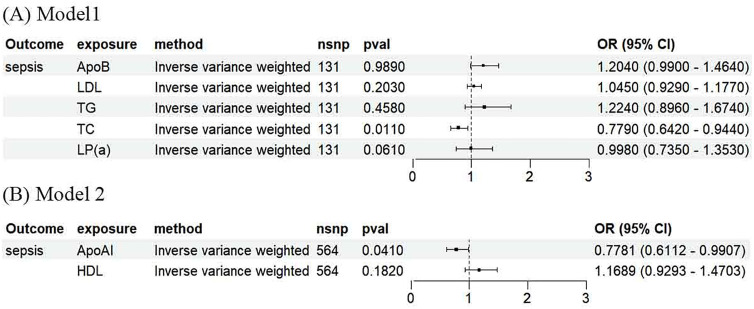
Multivariable Mendelian randomization. **(A)** Model 1 included apolipoprotein B, low-density lipoprotein cholesterol, triglycerides, total cholesterol, and lipoprotein A. **(B)** Model 2 included apolipoprotein A1 and high-density lipoprotein cholesterol. N SNP, number of single nucleotide polymorphisms; OR, odds ratio; CI, confidence interval; Apo, apolipoprotein; HDL-C, high-density lipoprotein cholesterol; LDL-C, low-density lipoprotein cholesterol; TG, triglycerides; TC, total cholesterol; Lp(a), lipoprotein A.

When the combined effects of ApoB, LDL-C, TG, TC, and Lp(a) were examined in Model 1 using the multivariable IVW method, in contrast to univariable MR, heightened levels of total cholesterol exhibited an association with lower risk of sepsis in multivariable MR (OR 0.779, 95% CI 0.642–0.944; P = 0.01) (Fig 3A). With the MR-Egger and MR-Lasso methods, the effect value was consistent with the IVW estimate (Supplement Table 4 in S2 File). No evidence of horizontal pleiotropy was detected (Supplement Table 5 in S2 File).

In a multivariable MR analysis incorporating ApoA-1 and HDL-C, increasing ApoA-I was associated with reduced risk of sepsis (OR 0.778, 95% CI 0.611–0.990; P = 0.04) (Fig 3B), which is consistent with the results of univariable MR. No causal effect was observed for any other variable under investigation. Remarkably, the robustness of the findings persisted following the application of both MR-Egger and MR-Lasso methods (Supplement Table 6 in S2 File). No indications of horizontal pleiotropy were detected (Supplement Table 7 in S2 File).

### Lipid-lowering drug targets and sepsis risk

**Fig 4:**
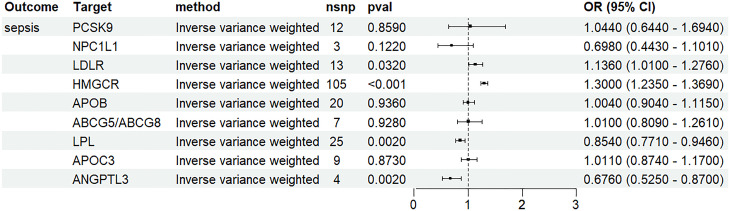
Association of genetically proxied drug targets with risk of sepsis. Data are represented as odds ratios (ORs) with 95% confidence intervals (error bars). OR, odds ratio; N SNP, single-nucleotide polymorphisms; HMGCR, HMG-CoA reductase; NPC1L1, Niemann-Pick C1-like protein 1; PCSK9, proprotein convertase subtilisin/kexin type 9; APOB, Apolipoprotein B-100; ABCG5, ATP Binding Cassette Subfamily G Member 5; ABCG8, ATP Binding Cassette Subfamily G Member 8; LDLR, LDL Receptor; ANGPTL3, angiopoietin-like 3; APOC3, Apolipoprotein C-III; LPL, lipoprotein lipase.

According to the primary pharmacological mechanism, these target genes were further classified as genes that lower LDL-C levels and genes that lower TG levels (Table 2). The genetic variants included in the drug-target analyses for each region can be found in Supplement Table 8 in S2 File. Associations for specific gene regions representing the targets of lipid-lowering drugs on sepsis are shown in Fig 4. SNPs that reduce the activity of ANGPTL3 had an obvious protective effect on sepsis in both the IVW method (OR 0.676, 95% CI 0.525–0.870; P = 0.002) and weighted median method (OR 0.664, 95% CI 0.501–0.881; P = 0.004). A similar result was noted for SNPs that reduce the activity of LPL regarding the protective effect against sepsis risk(IVW method: OR 0.854, 95% CI 0.771–0.946; P = 0.002. weighted median method: OR 0.852, 95% CI 0.743–0.978; P = 0.022.). Additionally, SNPs that reduce the activity of HMGCR and LDLR were associated with an increased risk of sepsis (OR 1.300, 95% CI 1.235–1.369; P = 1.38E-23), (OR 1.136, 95% CI 1.010–1.276; P = 0.032), while the inhibition of PCSK9 did not have a causal effect on sepsis risk (IVW method: p = 0.859). The results obtained through alternative MR methods were generally consistent (Supplement Table 9 in S2 File).

**Table 2:** Lipid-lowering drug classes, substances, and target genes.

pharmacologicalaction	Substance	Drug targets	Target genes	Gene region (GRCh37/hg19 by Ensembl)	SNP N
Reduced LDL-C		LDL Receptora	LDLR	chr19:11,200,038−11,244,492	13
	Pravastatin	HMG-CoA reductase	HMGCR	chr5:74,632,154−74,657,929	105
		Simvastatin			
		Lovastatin			
		Fluvastatin			
		Atorvastatin			
		Rosuvastatin			
	Ezetimibe	Niemann-Pick C1-like protein 1	NPC1L1	chr7:44,552,134−44,580,914	3
	Alirocumab	Proprotein convertase subtilisin/kexin type 9	PCSK9	chr1:55,505,221−55,530,525	12
		Evolocumab			
	Mipomersen	Apolipoprotein B-100	APOB	chr2:21,224,301−21,266,945	20
	Colesevelam	ATP Binding Cassette Subfamily G Member 5/8	ABCG5/ABCG8	chr2:44,039,611−44,066,004/chr2:44,066,103−44,105,605	14
		Colestipol			
		Cholestyramine			
Reduced TG		Lipoprotein Lipase	LPL	chr8:19,759,228−19,824,769	25
	Fenofibrate	Peroxisome proliferator-activated receptor-a	PPARA	chr22:46,546,424−46,639,653	0
		Gemfibrozil			
	Evinacumab	Angiopoietin-related protein 3	ANGPTL3	chr1:63,063,158−63,071,830	4
	Volanesorsen	Apolipoprotein C-III	APOC3	chr11:116,700,422−116,703,788	9

SNP N, number of single nucleotide polymorphisms; chr, chromosome; LDL-C, low-density lipoprotein cholesterol; TG, triglyceride.

## Discussion

In this MR study, we investigated the causal effect of genetically predicted lipid traits on sepsis using univariable MR and multivariable MR approaches. Our findings reveal a protective effect of increasing genetically predicted levels of HDL-C and ApoA-I on the risk of sepsis. Moreover, multivariable MR analysis showed that ApoA-I, ApoB, and LDL-C are causally associated with the risk of sepsis. Notably, we have identified ANGPTL3 and LPL as potential drug targets that significantly lowered the risk of sepsis. Our study provided strong evidence that ANGPTL3 and LPL are promising drug targets for sepsis.

In general, we have verified the existence of a positive causal relationship between dyslipidemias and sepsis, which is consistent with the findings of previous investigations. Our findings show a causal relationship between elevated HDL-C levels and reduced risk of sepsis. Two extensive prospective cohorts have demonstrated that an approximate 30% reduction in HDL levels occurs in patients with sepsis on the day of hospital admission compared with healthy controls [22,23]. Decreased levels of HDL are associated with heightened sepsis mortality rates and can serve as prognostic indicators for multiorgan dysfunction [24–27]. Furthermore, a prospective study by Lekkou A. et al. reveals that serum HDL consistently diminishes in sepsis non-survivors on day 0, day 3, day 7, and day 10 in comparison to survivors [26]. Septic patients who present with HDL concentrations below 25.1 mg/dl upon hospital admission exhibit susceptibility to adverse outcomes, including the necessity for intensive care unit care, development of multiple or single organ dysfunctions, and mortality [25].

Plasma contains about 90–95% of ApoA-I bound to HDL particles [28]. Previous research have concluded that HDL directly affects the inflammation process [29–31]. It has been shown that HDL, especially ApoA-I, can neutralize Lipopolysaccharide(LPS) by increasing liver biliary excretion and clearance, thereby preventing septic shock [32]. Clinical studies have emphasized that during sepsis, levels of HDL-C decrease [33,34], which is associated with increased mortality and adverse clinical outcomes [35]. The anti-inflammatory effects of HDL apparently play a potentially important role in infectious diseases. A recent multicentre prospective study by M. Trinder et al. confirmed a potential causal relationship between HDL and sepsis [36]. This study concluded that for each 1 mmol/L increase in HDL levels, the risk of infectious injury decreased by 0.84. Furthermore, using Mendelian randomization with HDL-C-related genetic variants further demonstrated a causal relationship between increased HDL-C and reduced risk of infection-related hospitalization, as well as a significant negative correlation between HDL-C and sepsis mortality rates [36]. This is consistent with our research findings, which indicate a causal relationship between high-density lipoprotein and sepsis.

Extensive studies have consistently highlighted low cholesterol levels as a risk factor for sepsis [27,37–39]. We observed an inverse relationship between elevated total cholesterol levels and the risk of sepsis. These findings are also supported by Guirgis FW et al. and Liang et al., who independently concluded that increased cholesterol levels are protective against the risk of sepsis [40,41]. Interestingly, our study did not uncover a causal association between lower LDL-C and sepsis risk. Walley KR et al. also argue that low LDL concentrations in sepsis may be more associative with rather than causal of increased mortality risk [42].

It has been proposed that pharmacological agents targeting lipid metabolism could have a preventive effect on sepsis. Liappis AP et al. reported that statin therapy resulted in decreased mortality rates among patients with sepsis [43]. Almog Y et al. further demonstrated a correlation between prior statin therapy and a reduction of severe sepsis [44]. In line with these findings, two studies conducted by Merx MW et al. exhibited increased survival rates in murine models of sepsis after simvastatin treatment [45,46]. However, our study found that inhibitors of HMGCR increased the risk of sepsis. A cohort study published in The Lancet has reported that statins increase the risk of type 2 diabetes and body weight, which are considered risk factors for sepsis [47]. This might be the main reason for this result. Additionally, only TG-lowering genetic variants in ANGPTL3 and LPL were associated with lower sepsis risk in our study. On the contrary, we did not find significant associations between TG levels and sepsis when comparing the effects of the TG-lowering drugs such as fenofibrate, gemfibrozil and evinacumab. Therefore a reduction in sepsis cannot be achieved only by using drugs to reduce TG levels. PCSK9 inhibitors are currently under investigation as lipid-lowering agents. Walley KR, et al. have proposed that inhibition of PCSK9 leads to diminished production of inflammatory cytokine and attenuated physiological responses to endotoxin in septic mice [42,48]. Nonetheless, our study failed to provide evidence supporting the conclusion that PCSK9 inhibition can reduce the risk of sepsis. Interestingly, Natasja de Bont et al. discovered that low-density lipoprotein receptor knock-out mice were protected against lethal endotoxemia and gram-negative infections [49]. Our results are consistent with this finding, showing that genetic mimicry of LDLR heightens the susceptibility to sepsis. This observation further elucidated the potential causal association. A growing body of genome-wide association studies (GWAS) has consistently identified a positive association between elevated plasma triglyceride levels and non-alcoholic fatty liver disease (NAFLD). However, recent drug-target association studies have revealed that among multiple triglyceride-lowering therapeutic targets—including PPARA, ANGPTL3, ANGPTL4, APOC3, and LPL—only lipoprotein lipase (LPL) activation reduces the risk of NAFLD. This suggests that selectively targeting LPL may represent a unique therapeutic strategy for mitigating metabolic dysfunction-associated steatotic liver disease (MASLD) [50,51].

Furthermore, a deeper exploration of mechanistic pathways within broader drug categories (e.g., triglyceride modulators), supported by metabolomics profiling, could enhance our understanding of underlying biological mechanisms. Such insights may refine drug development strategies, enabling more precise and effective interventions to improve clinical outcomes.

## Limitation

We acknowledge certain constraints in our study. Primarily, the scope of our findings was limited to individuals of European descent, therefore precautions should be exercised in extrapolating these outcomes to other ethnic groups. Furthermore, we only used genetic data to find a possible causal relationship between lipid characteristics and sepsis, the mechanism of which is yet uncertain and still requires further confirmation. Lastly, our study only predicts the on-target effects of specific pharmacological targets, with these models do not estimate potential off-target effects.

## Conclusion

In summary, a potential causal association was observed between elevated concentrations of ApoA-1, HDL, and TC and a diminished susceptibility to sepsis. Moreover, this study demonstrates that ANGPTL3 and LPL are promising candidate drug targets for the treatment of sepsis.

## Supporting information

S1 File Supplementary Figure 1–14. (DOCX)

S2 File Supplementary Table 1–9. (XLSX)

S1 Data STROBE-MR checklist. (XLSX)
