# Supplementary material for: Genetic association of lipids characteristics and lipid lowering drug target genes with sepsis
Source: PLoS One. 2025 Sep 11;20(9):e0331023. doi: 10.1371/journal.pone.0331023 (PMC12425328; doi:10.1371/journal.pone.0331023)

# Supplementary Figures

### **Supplementary** **Figure 1.** Scatter plots showing the effect of ApoA-1 on the outcome via Inverse variance weighted, MR - Egger, weighted median, weighted mode, and simple mode method.

1. (b)


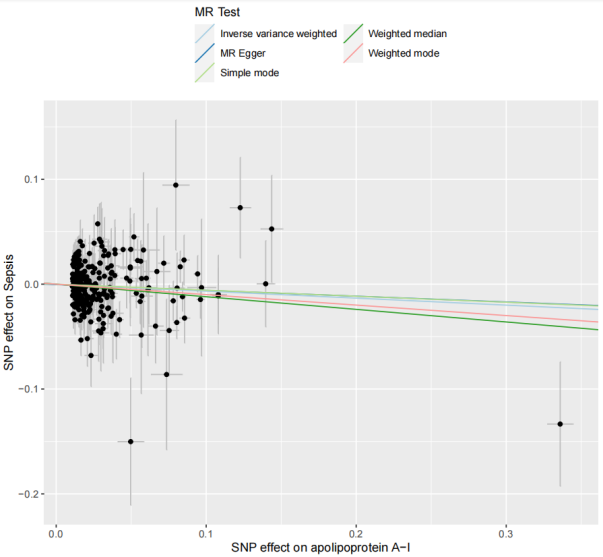

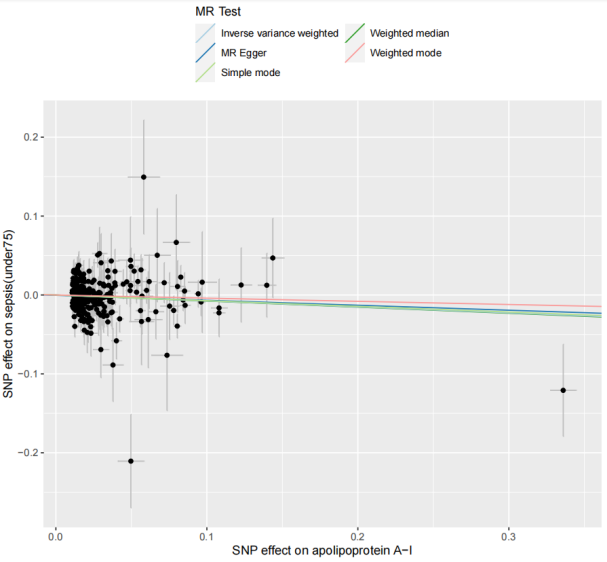


(c) (d)


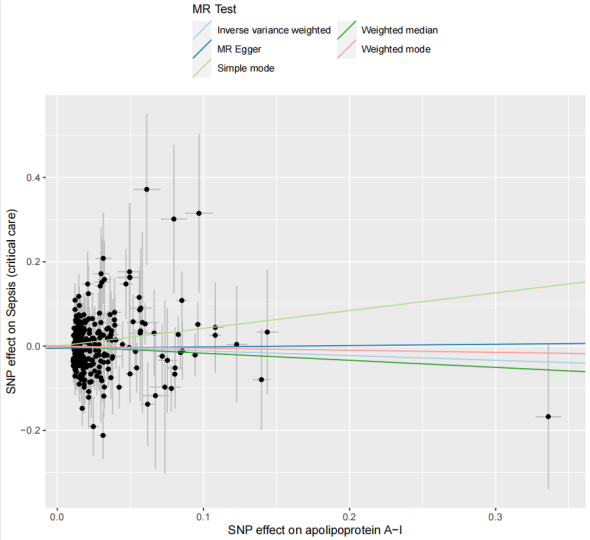

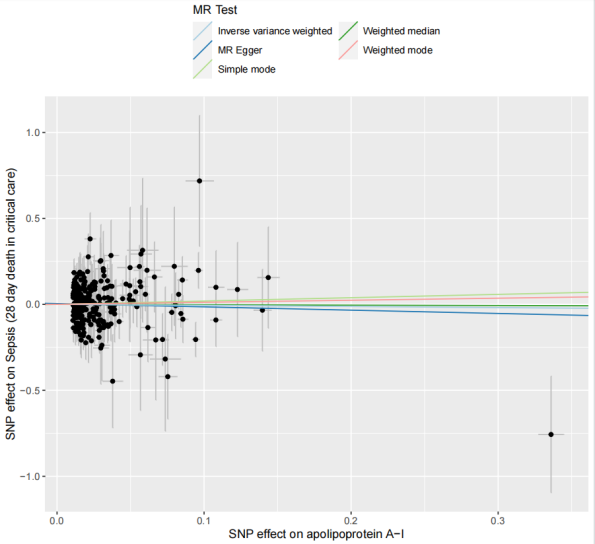


1. the effect of ApoA-1 on sepsis. (b) the effect of ApoA-1 on sepsis (under 75). (c) the effect of ApoA-1 onsepsis (critical care). (d) the effect of ApoA-1 on sepsis (28 day death in critical care).

### **Supplementary Figure 2.** Scatter plots showing the effect of ApoB on the outcome via Inverse variance weighted, MR - Egger, weighted median, weighted mode, and simple mode method.

1. (b)


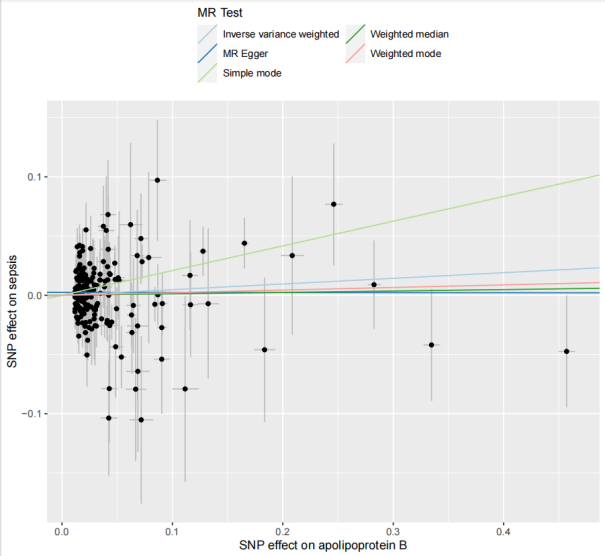

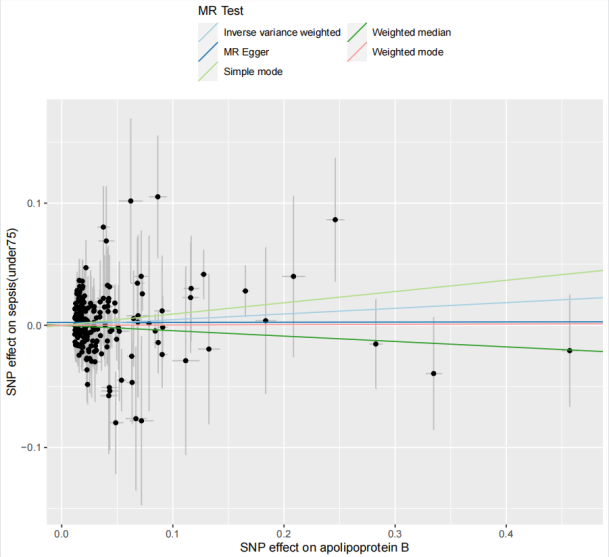


(c) (d)


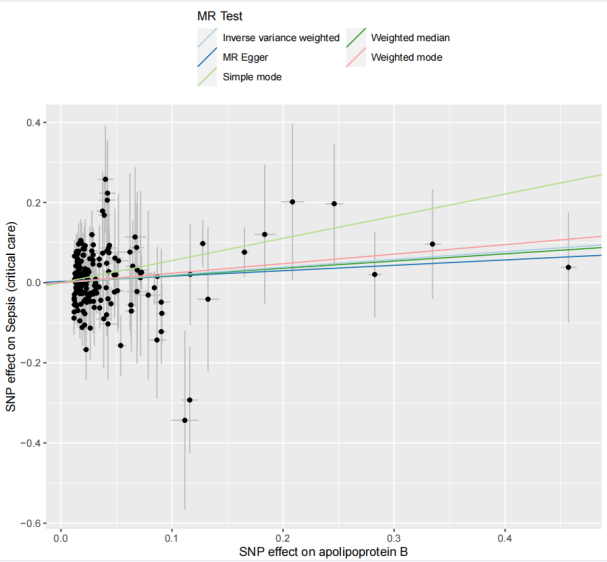

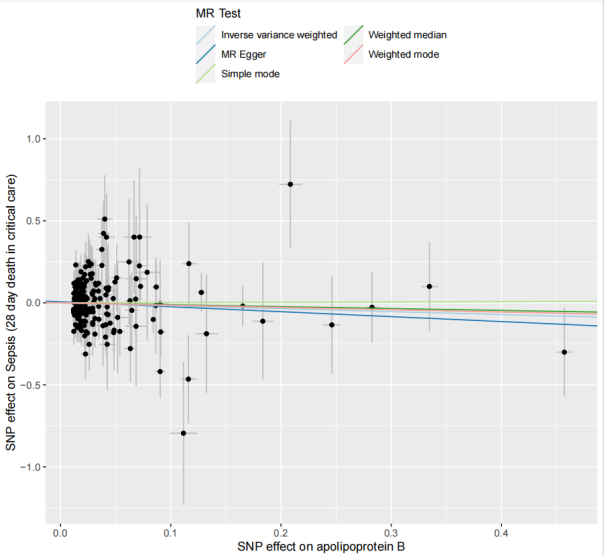


1. the effect of ApoB on sepsis. (b) the effect of ApoB on sepsis (under 75). (c) the effect of ApoB onsepsis (critical care). (d) the effect of ApoB on sepsis (28 day death in critical care).

### **Supplementary Figure 3.** Scatter plots showing the effect of HDL on the outcome via Inverse variance weighted, MR - Egger, weighted median, weighted mode, and simple mode method.

1. (b)


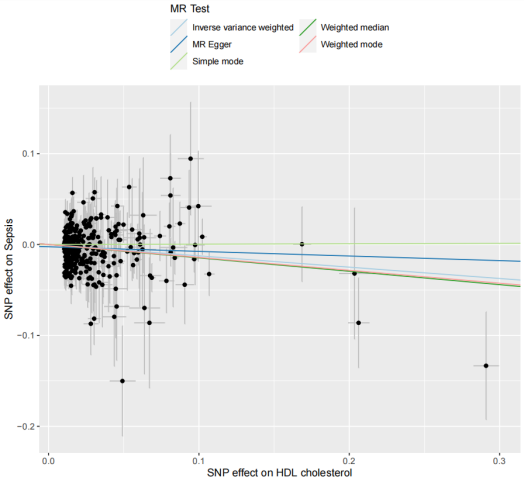

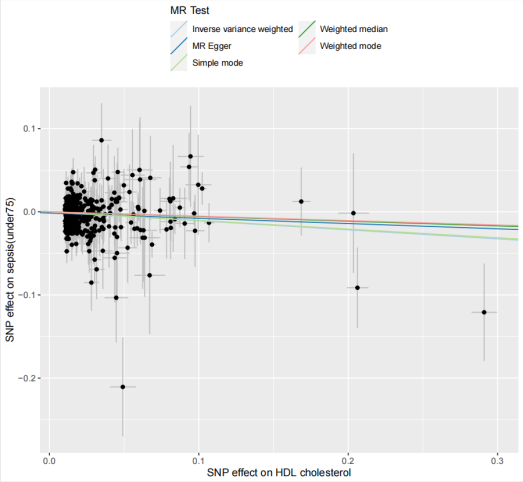


(c) (d)


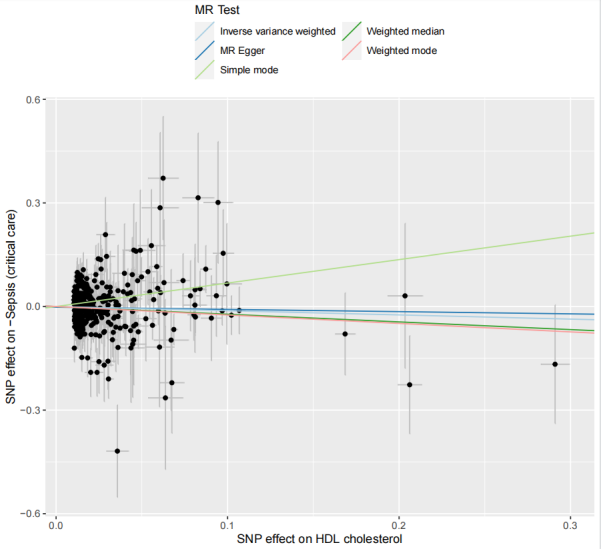

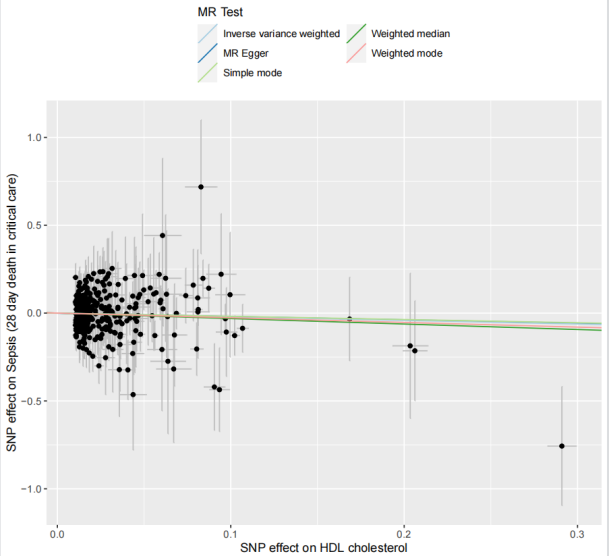


1. the effect of HDL on sepsis. (b) the effect of HDL on sepsis (under 75). (c) the effect of HDL onsepsis (critical care). (d) the effect of HDL on sepsis (28 day death in critical care).

### **Supplementary Figure 4.** Scatter plots showing the effect of LDL-C on the outcome via Inverse variance weighted, MR - Egger, weighted median, weighted mode, and simple mode method.

1. (b)


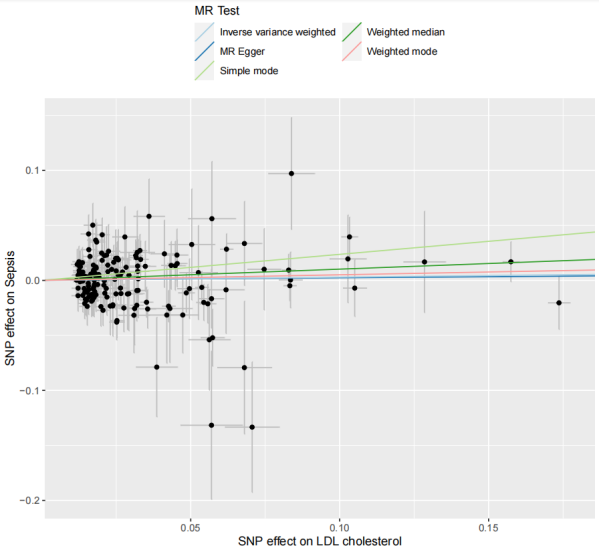

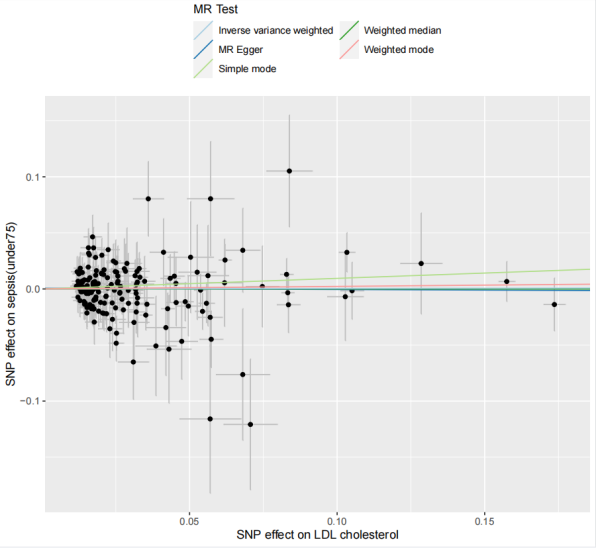


(c) (d)


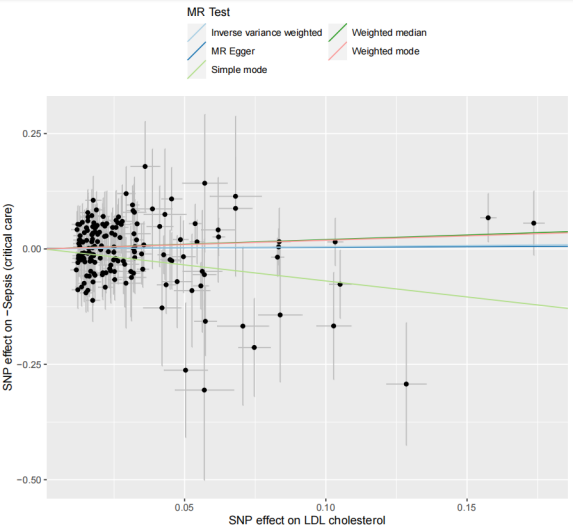

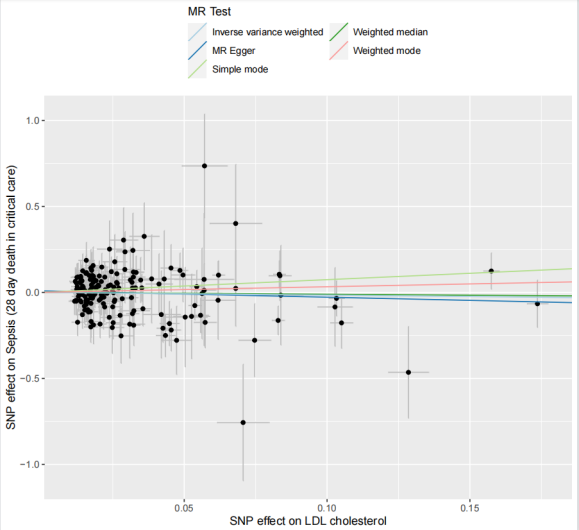


1. the effect of LDL-C on sepsis. (b) the effect of LDL-C on sepsis (under 75). (c) the effect of LDL-C onsepsis (critical care). (d) the effect of LDL-C on sepsis (28 day death in critical care).

### **Supplementary Figure 5.** Scatter plots showing the effect of TC on the outcome via Inverse variance weighted, MR - Egger, weighted median, weighted mode, and simple mode method.

1. (b)


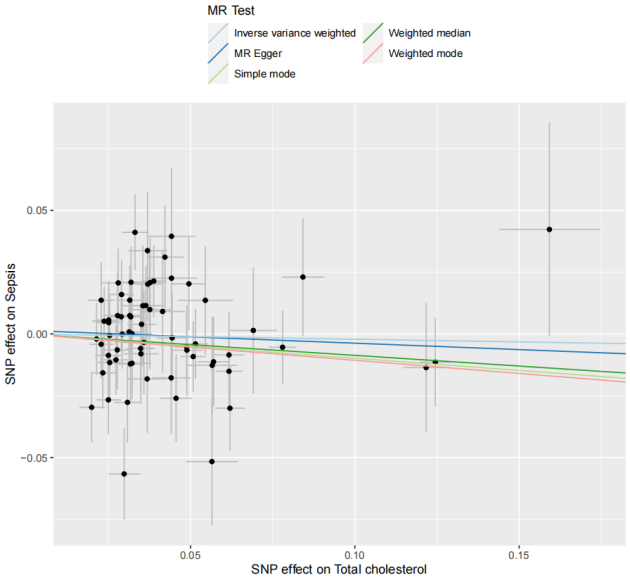

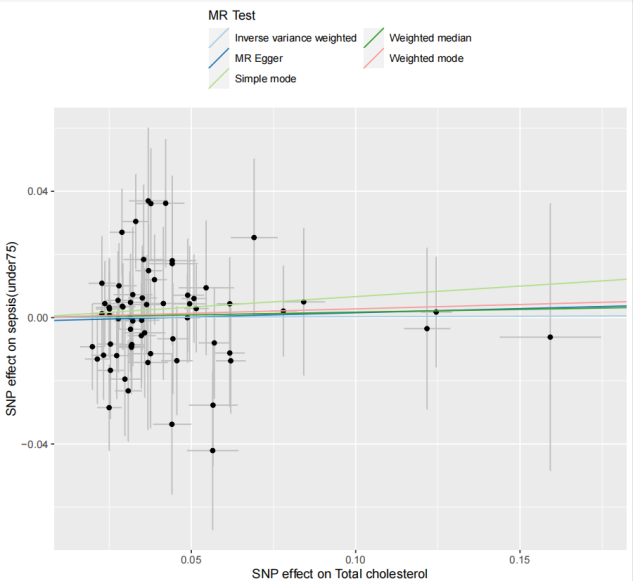


(c) (d)


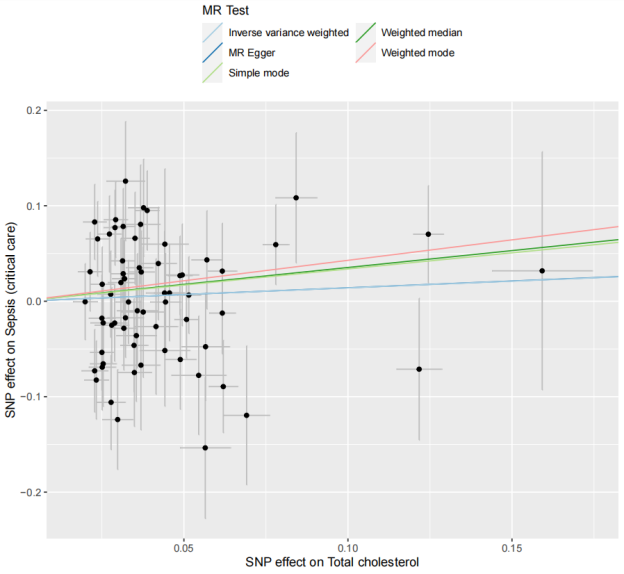

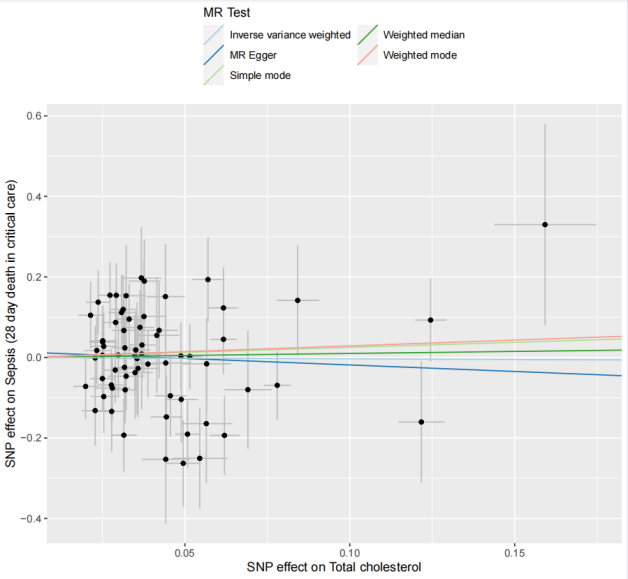


1. the effect of TC on sepsis. (b) the effect of TC on sepsis (under 75). (c) the effect of TC onsepsis (critical care). (d) the effect of TC on sepsis (28 day death in critical care).

### **Supplementary Figure 6.** Scatter plots showing the effect of TG on the outcome via Inverse variance weighted, MR - Egger, weighted median, weighted mode, and simple mode method.

1. (b)


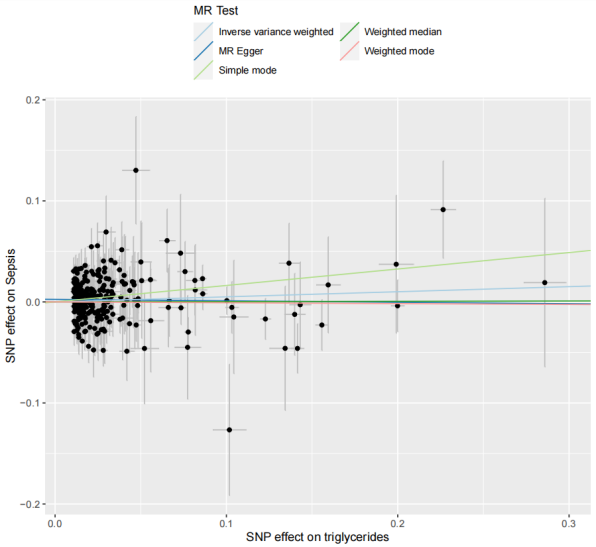

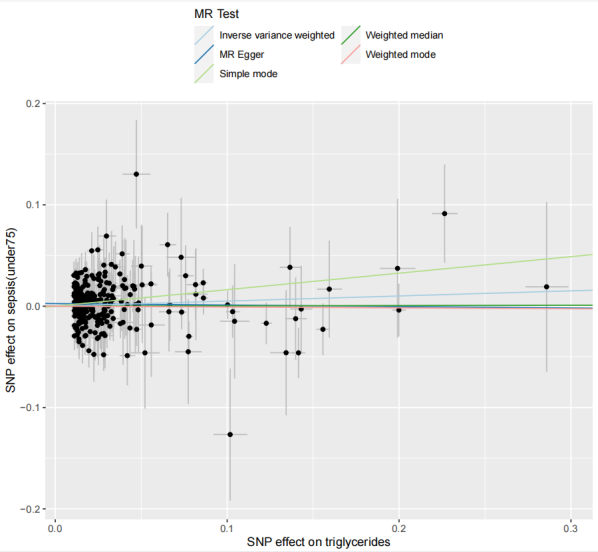


(c) (d)


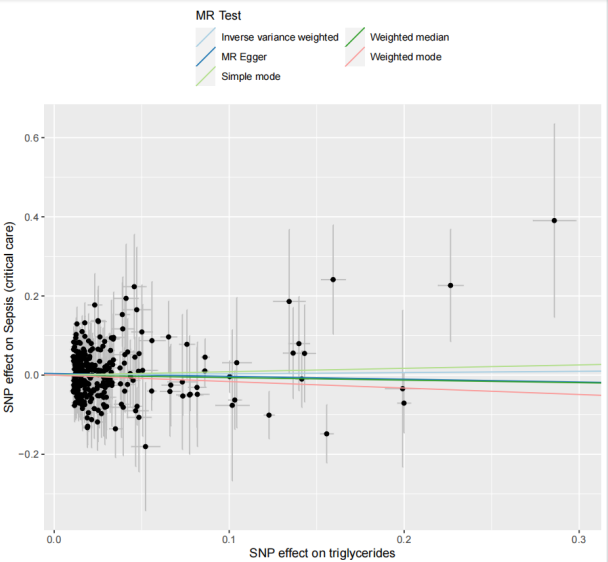

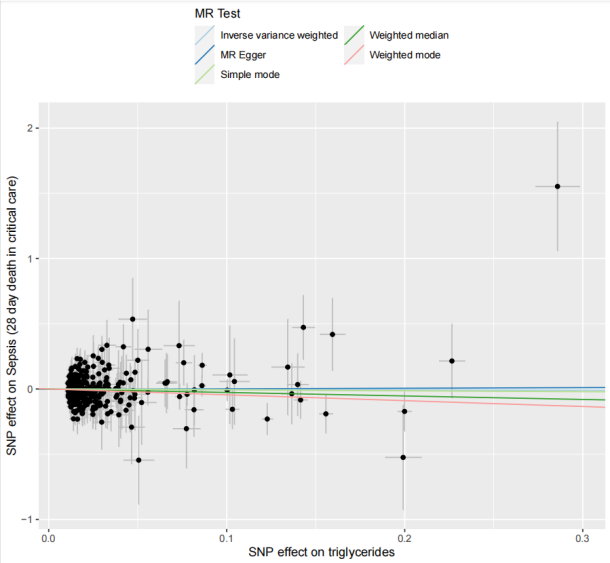


1. the effect of TG on sepsis. (b) the effect of TG on sepsis (under 75). (c) the effect of TG onsepsis (critical care). (d) the effect of TG on sepsis (28 day death in critical care).

### **Supplementary Figure 7.** Scatter plots showing the effect of Lp(a) on the outcome via Inverse variance weighted, MR - Egger, weighted median, weighted mode, and simple mode method.

1. (b)


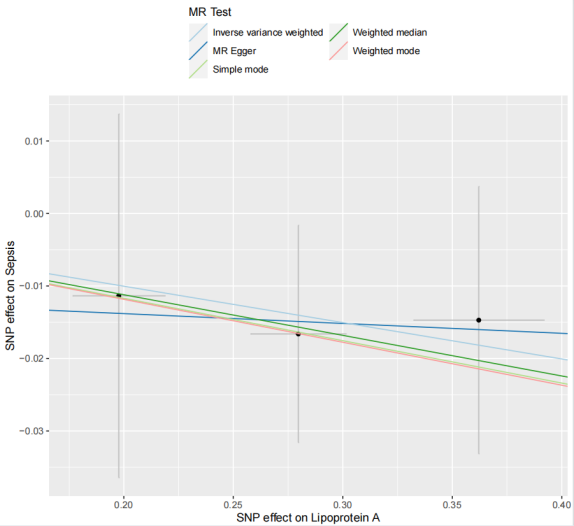

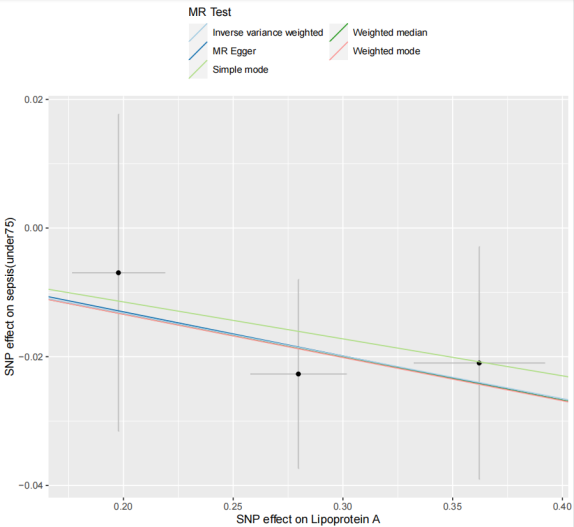


(c) (d)


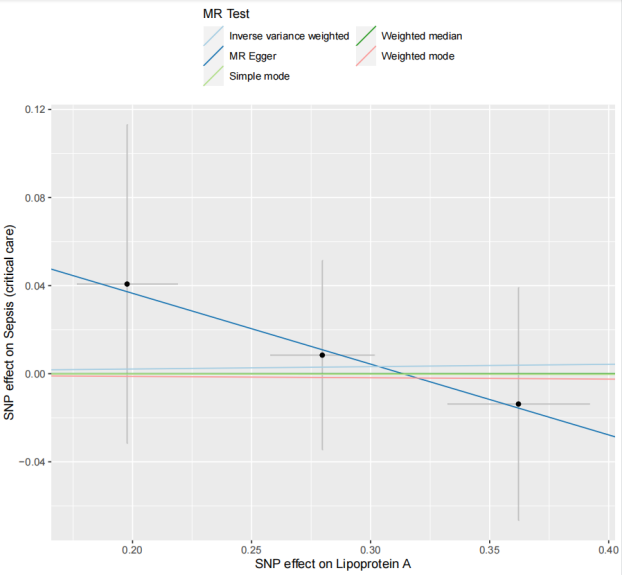

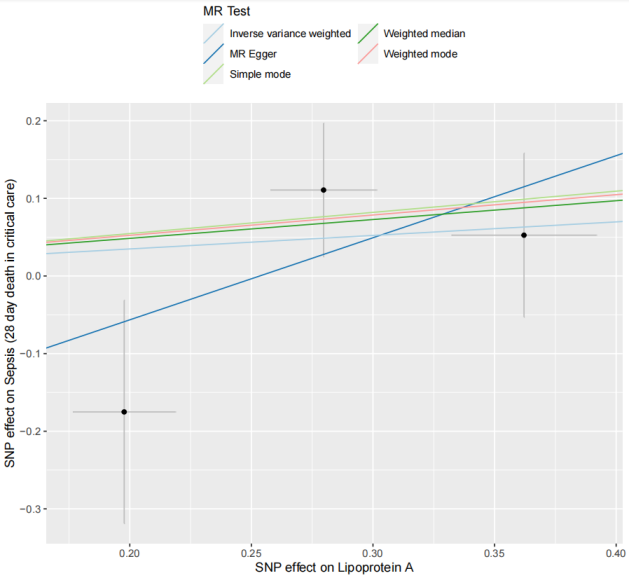


1. the effect of Lp(a) on sepsis. (b) the effect of Lp(a) on sepsis (under 75). (c) the effect of Lp(a) on sepsis (critical care). (d) the effect of Lp(a) on sepsis (28 day death in critical care).

**Supplementary Figure 8.** Plots of “leave-one-out” analyses for MR analyses of the causal effect of ApoA-1 on the outcome.

1. the effect of ApoA-1 on sepsis.


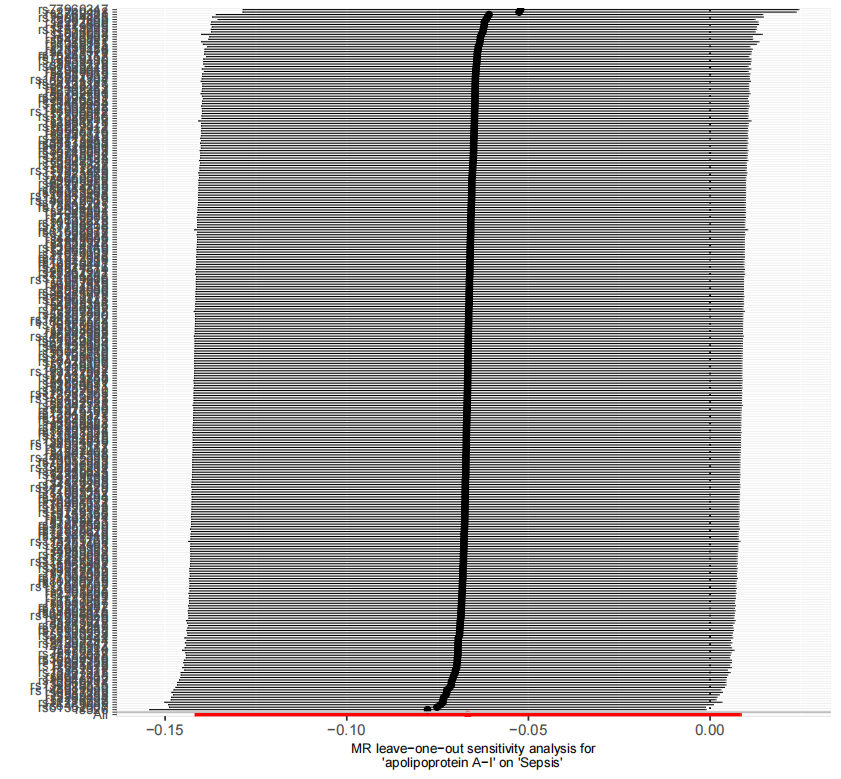


1. the effect of ApoA-1 on sepsis (under 75).


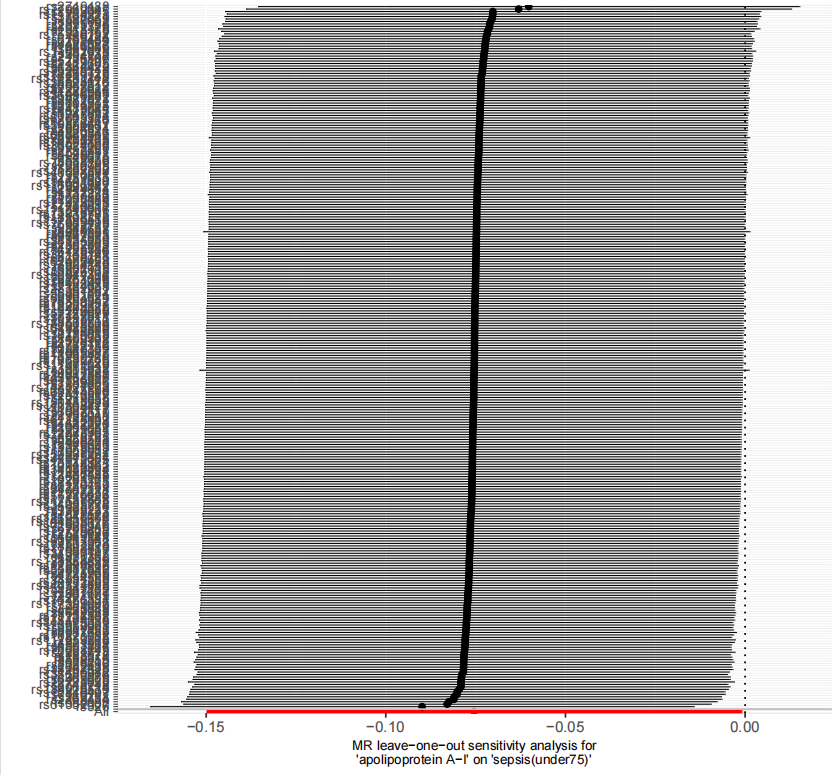


1. the effect of ApoA-1 on sepsis (critical care).


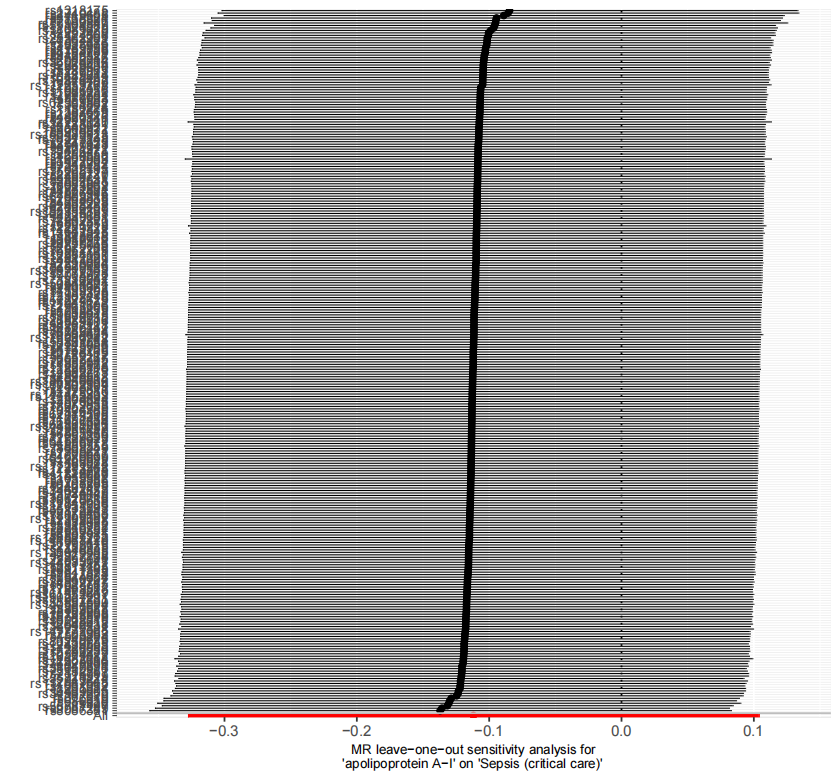


1. the effect of ApoA-1 on sepsis (28 day death in critical care).


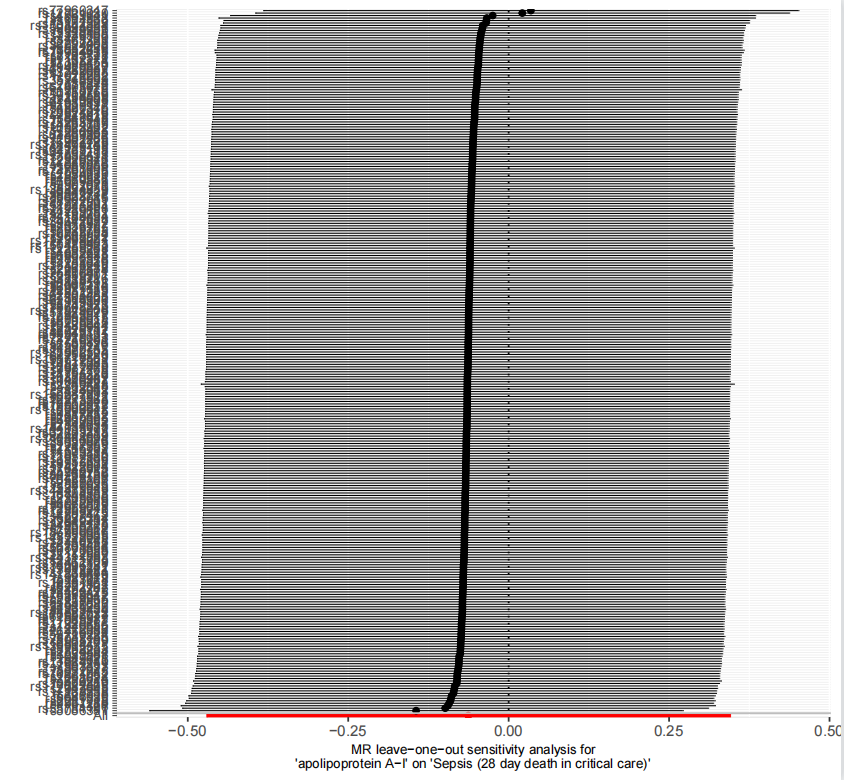


**Supplementary Figure 9.** Plots of “leave-one-out” analyses for MR analyses of the causal effect of ApoB on the outcome.

1. the effect of ApoB on sepsis.


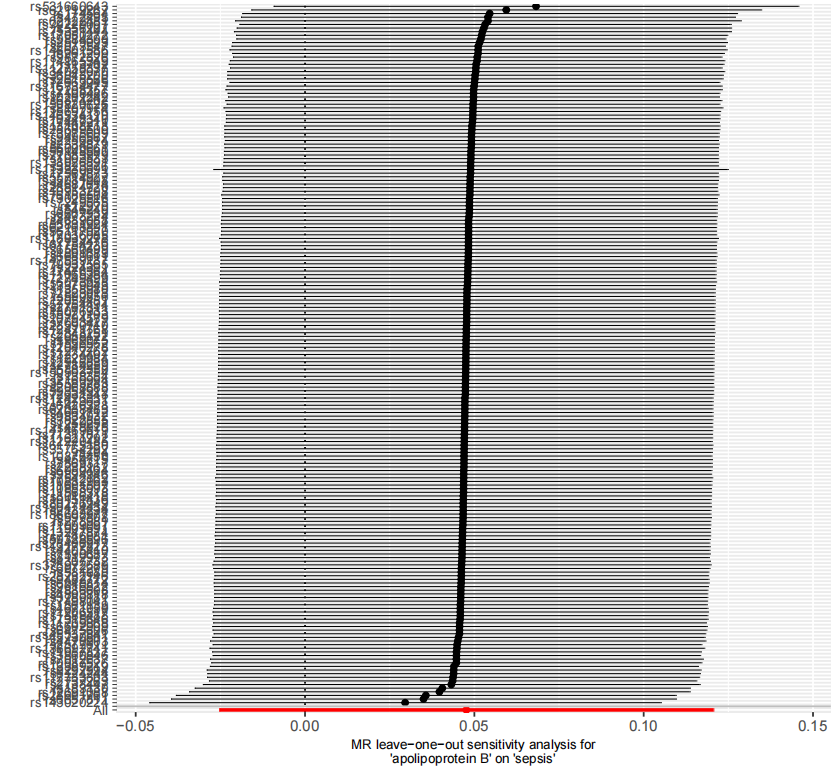


1. the effect of ApoB on sepsis (under 75).


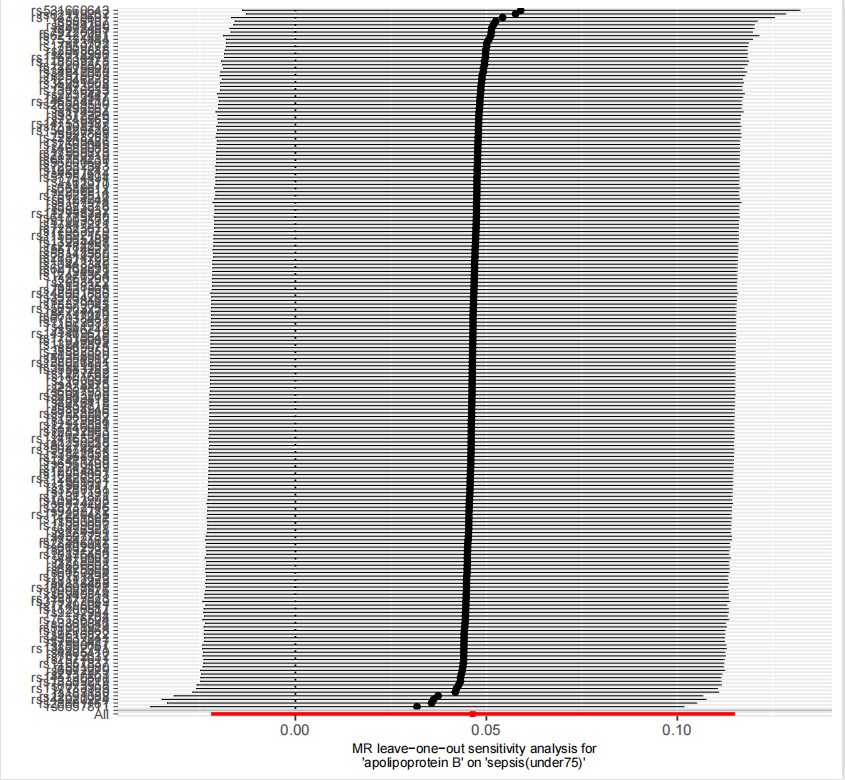


1. the effect of ApoB on sepsis (critical care).


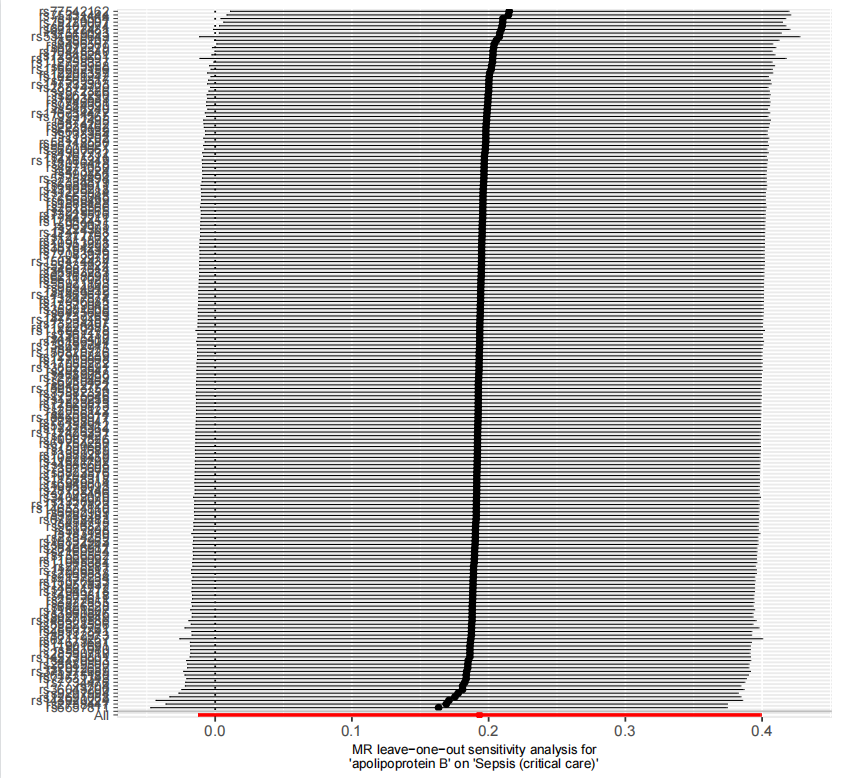


1. the effect of ApoB on sepsis (28 day death in critical care).


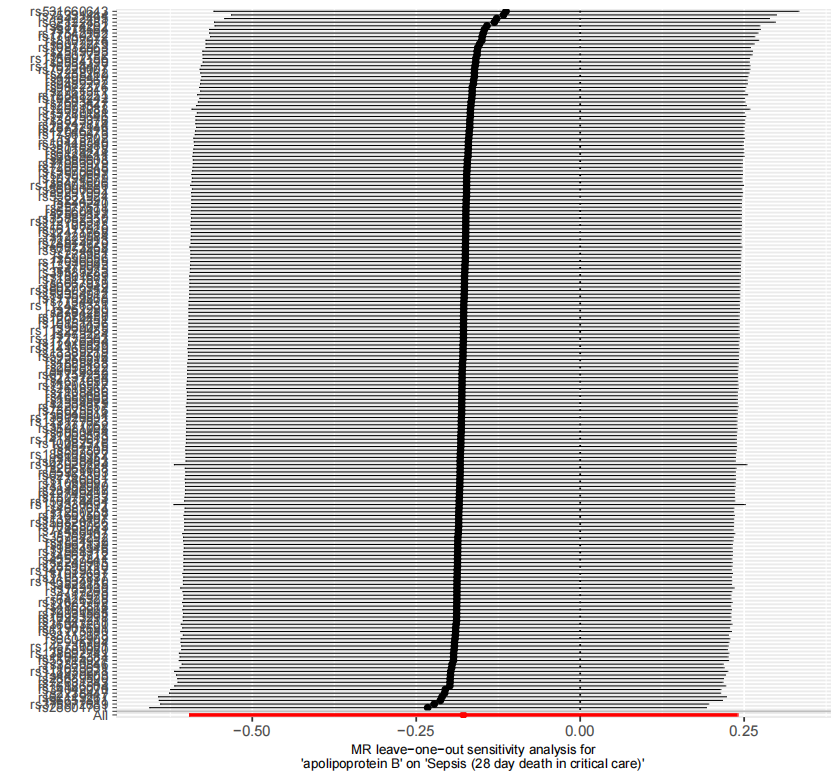


**Supplementary Figure 10.** Plots of “leave-one-out” analyses for MR analyses of the causal effect of HDL on the outcome.

1. the effect of HDL on sepsis.


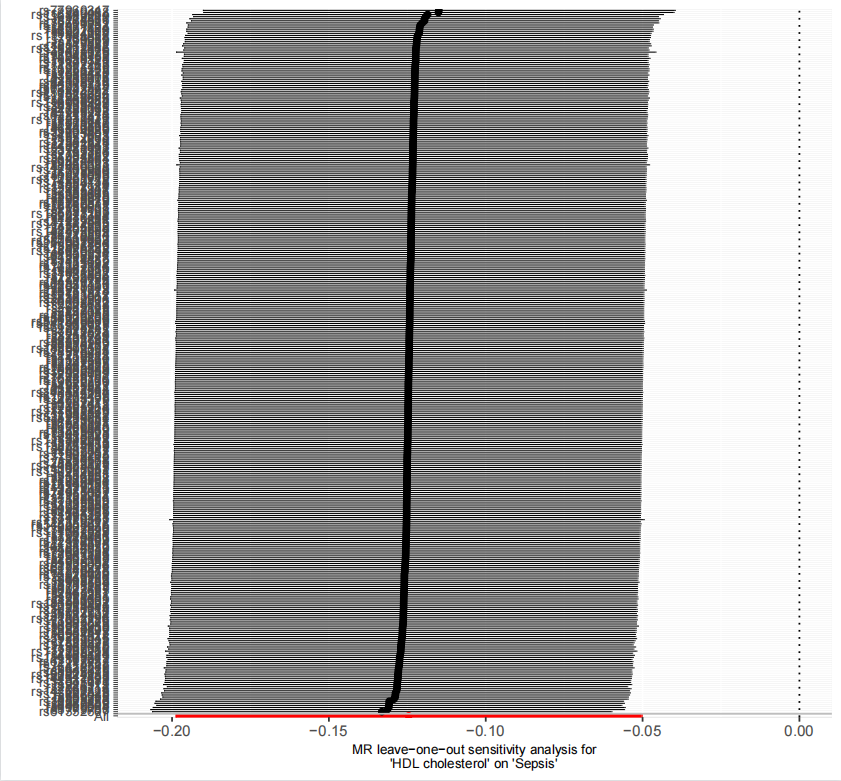


1. the effect of HDL on sepsis (under 75).


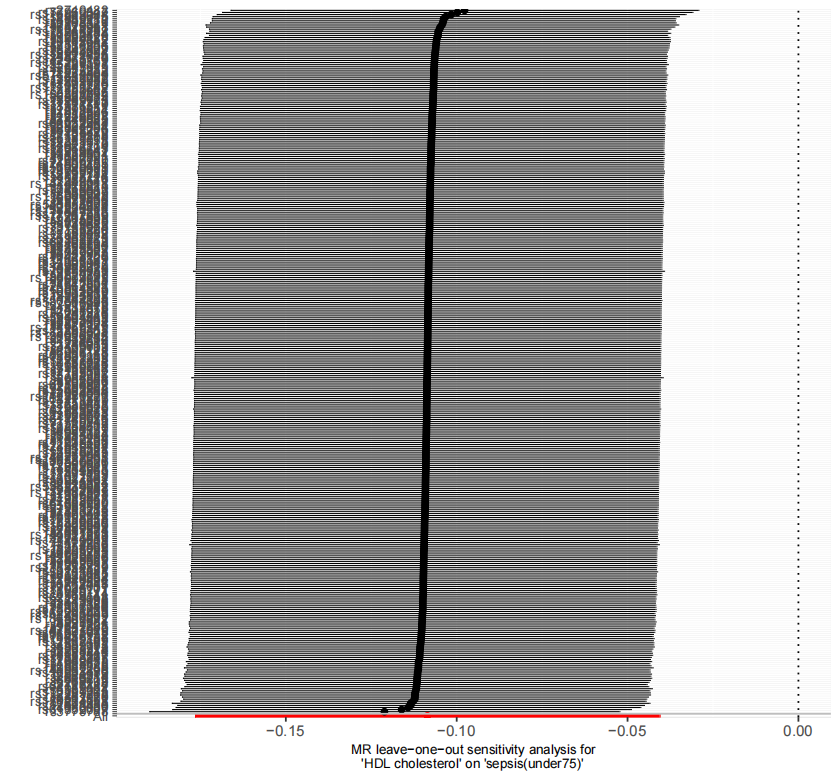


1. the effect of HDL on sepsis (critical care).


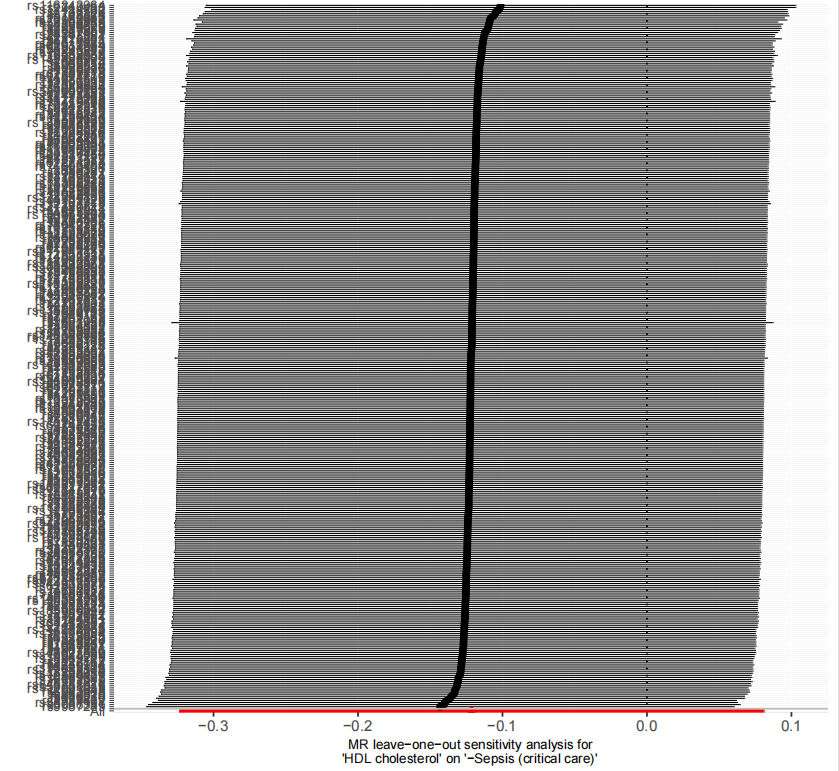


1. the effect of HDL on sepsis (28 day death in critical care).


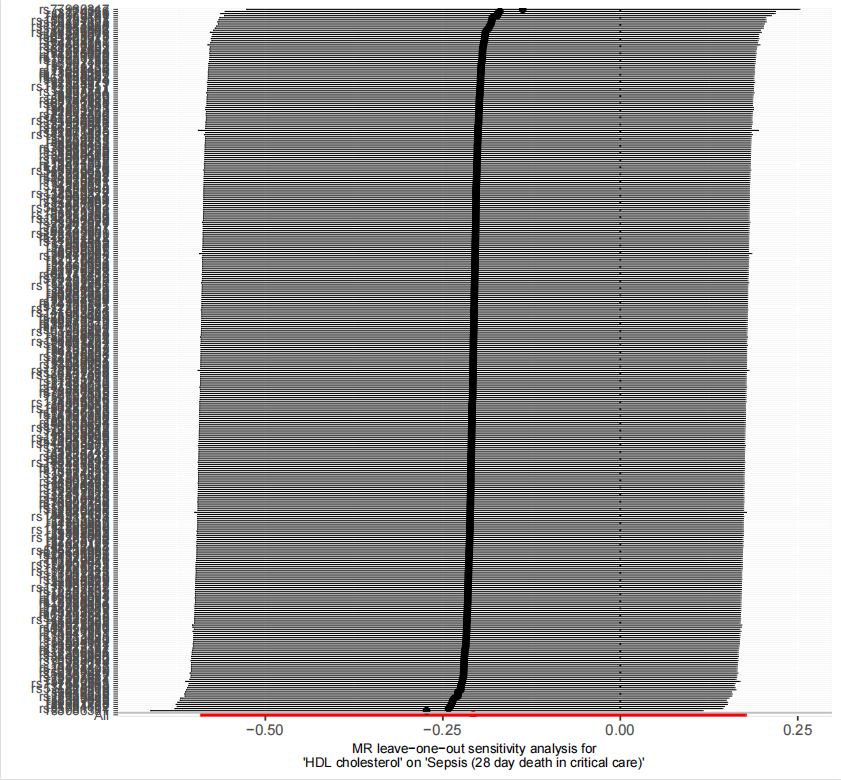


**Supplementary Figure 11.** Plots of “leave-one-out” analyses for MR analyses of the causal effect of LDL-C on the outcome.

1. the effect of LDL-C on sepsis.


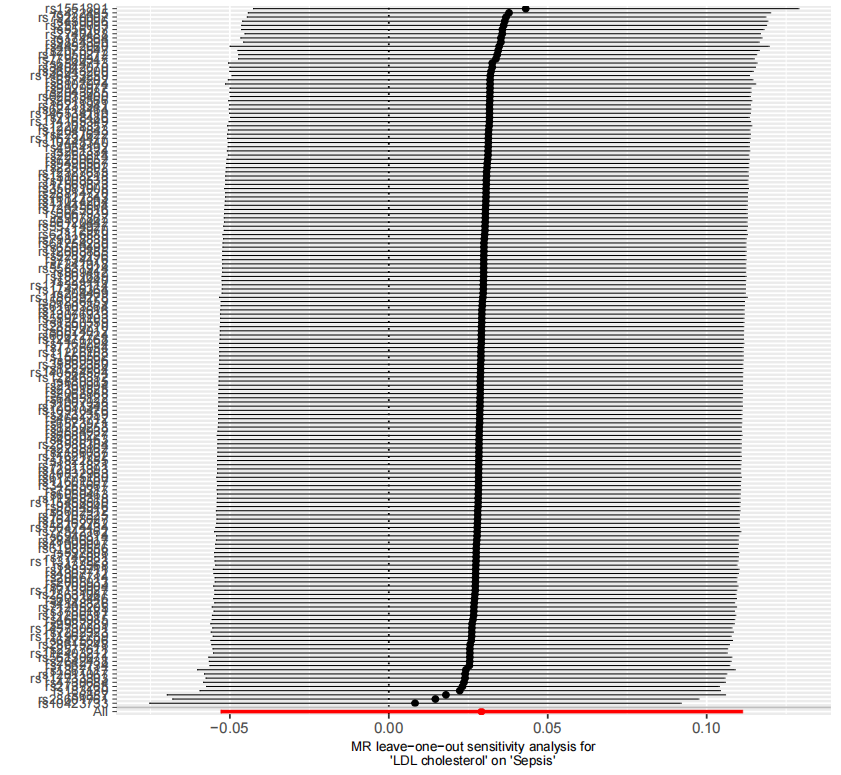


1. the effect of LDL-C on sepsis (under 75).


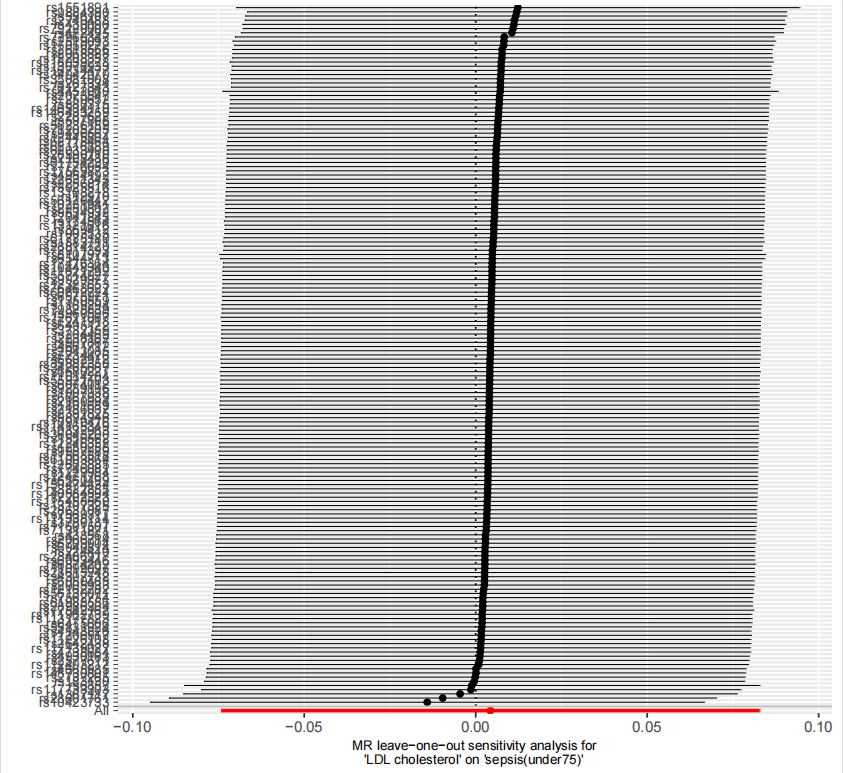


1. the effect of LDL-C on sepsis (critical care).


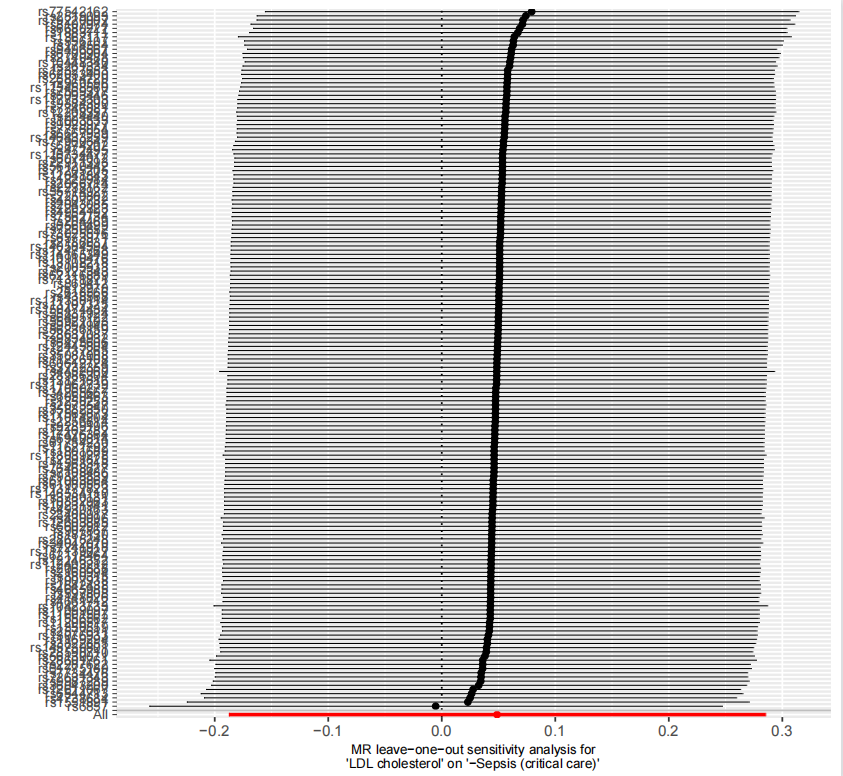


1. the effect of LDL-C on sepsis (28 day death in critical care).


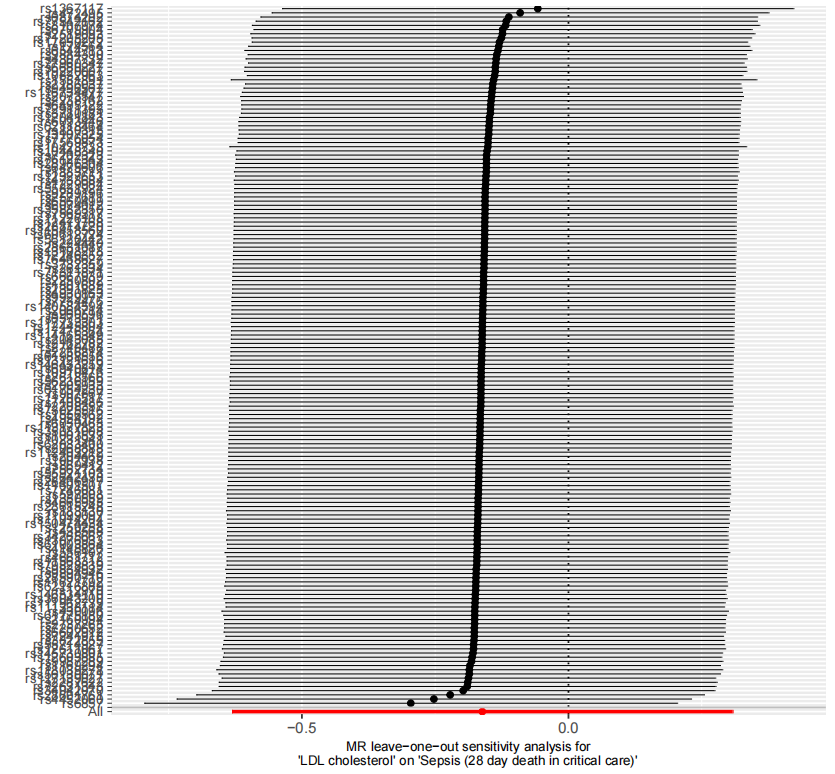


**Supplementary Figure 12.** Plots of “leave-one-out” analyses for MR analyses of the causal effect of TC on the outcome.

1. the effect of TC on sepsis.


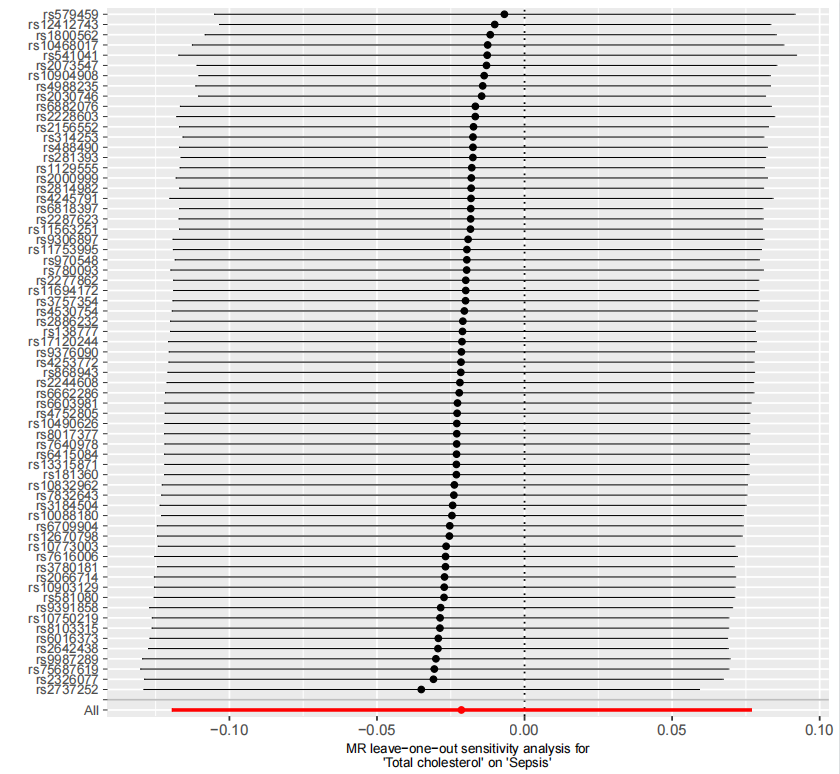


1. the effect of TC on sepsis (under 75).


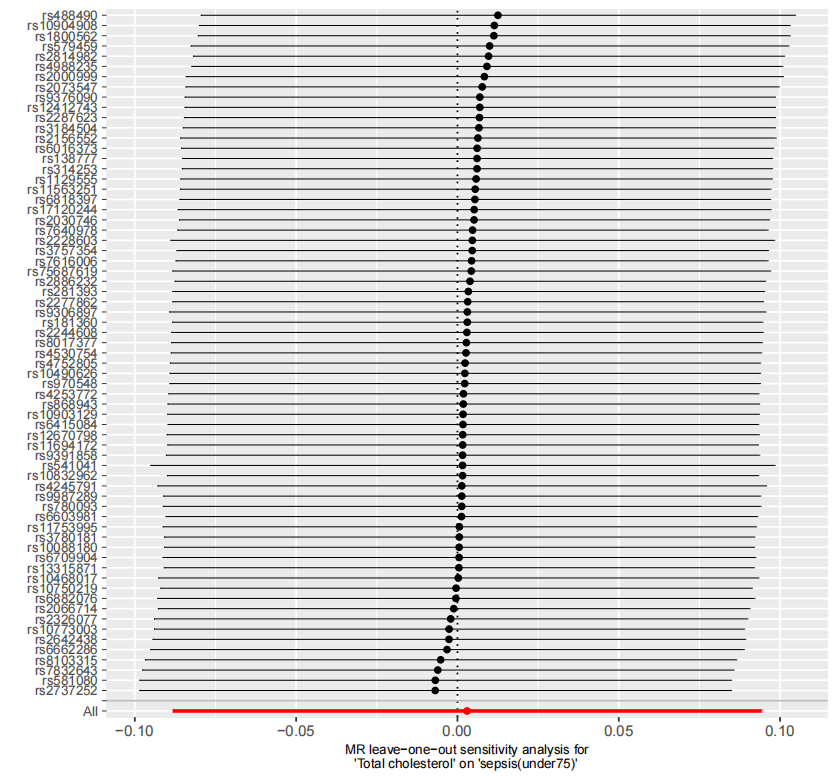


1. the effect of TC on sepsis (critical care).


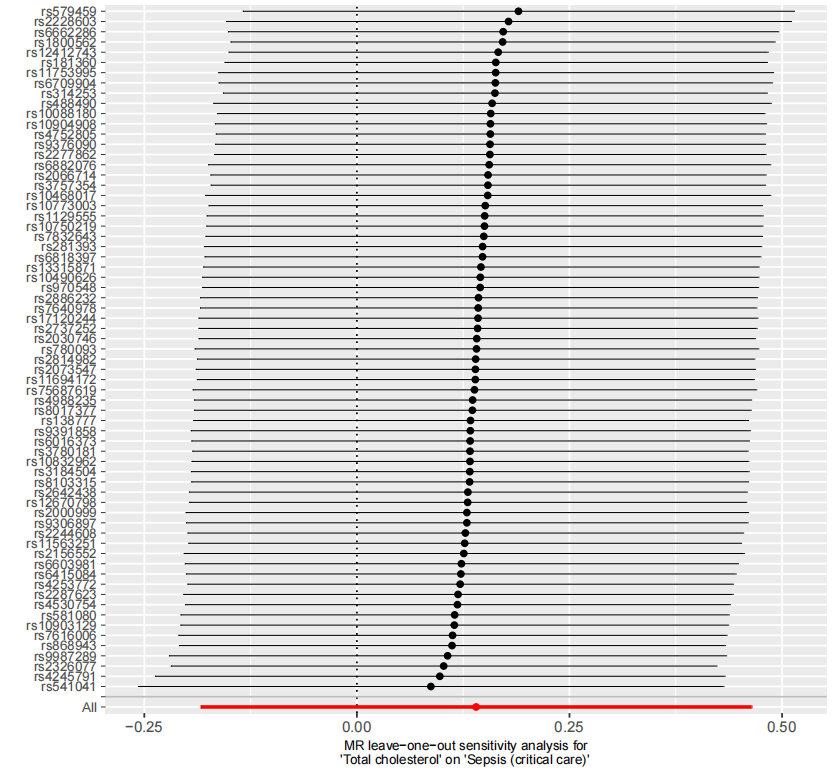


1. the effect of TC on sepsis (28 day death in critical care).


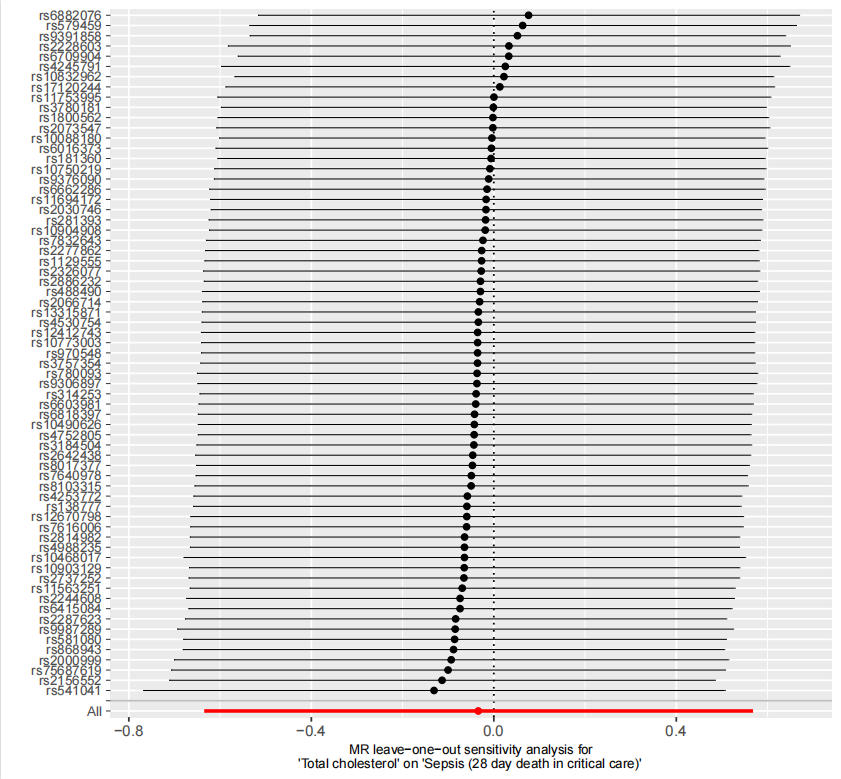


**Supplementary Figure 13.** Plots of “leave-one-out” analyses for MR analyses of the causal effect of TG on the outcome.

1. the effect of TG on sepsis.


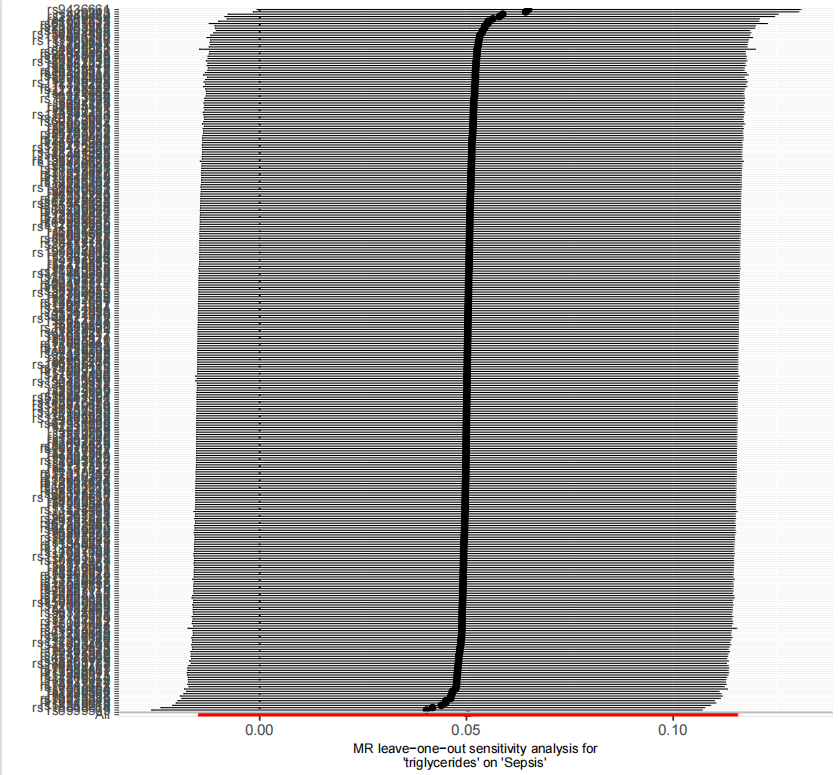


1. the effect of TG on sepsis (under 75).


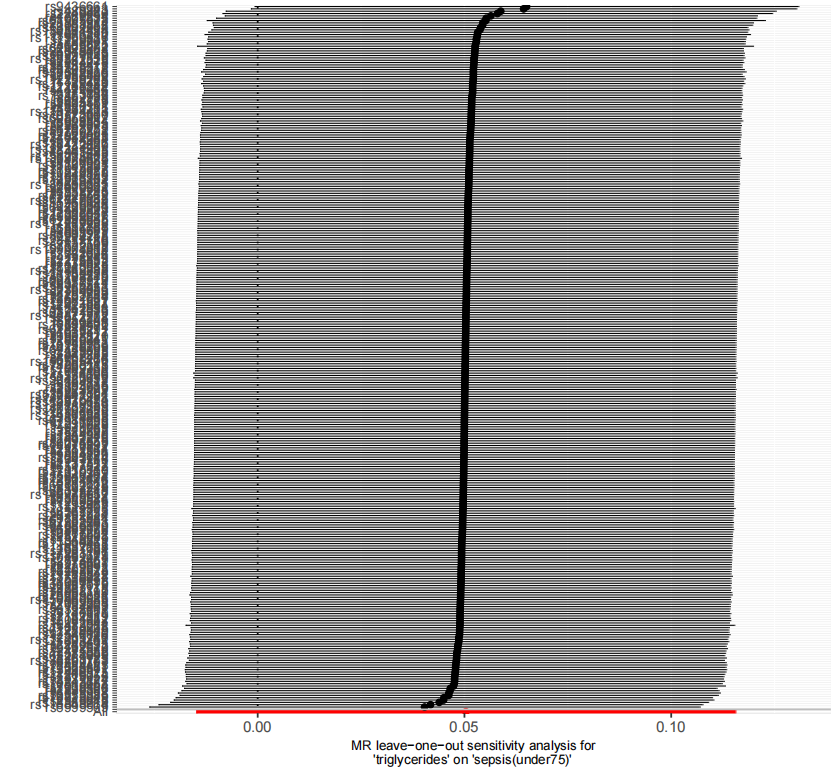


1. the effect of TG on sepsis (critical care).


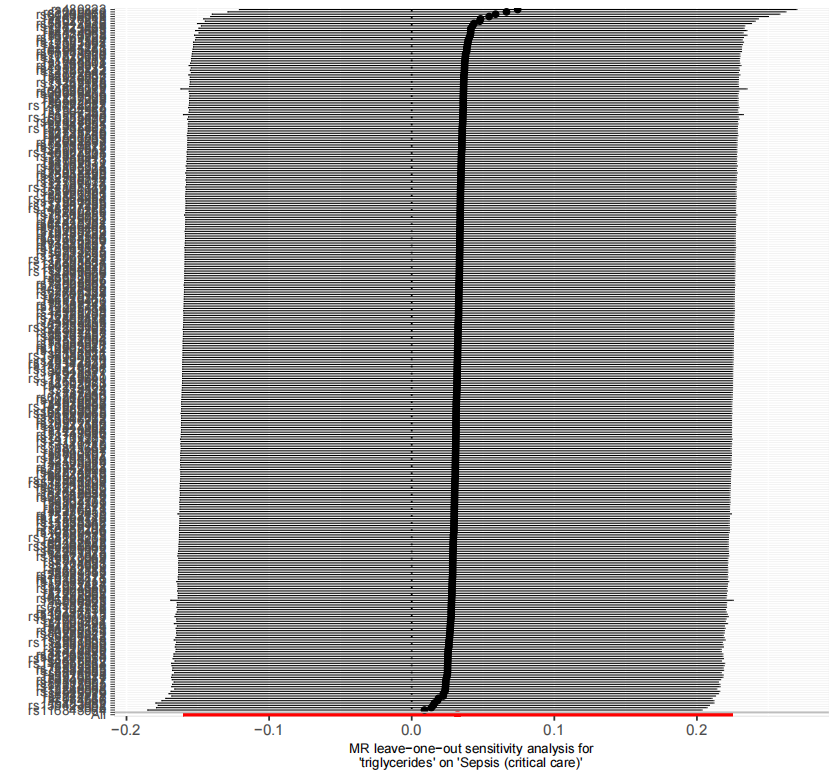


1. the effect of TG on sepsis (28 day death in critical care).


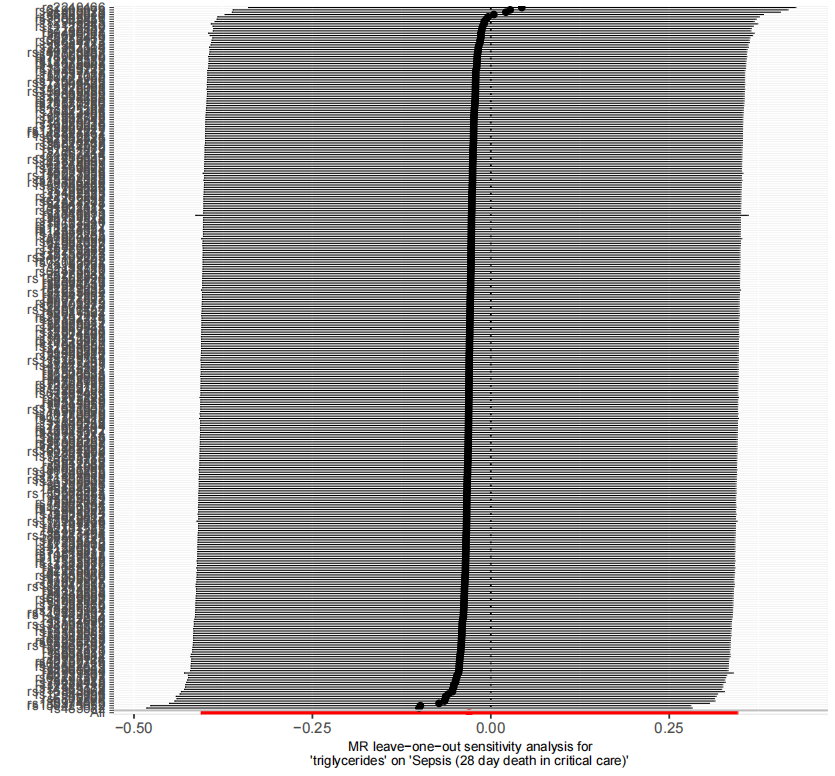


**Supplementary Figure 14.** Plots of “leave-one-out” analyses for MR analyses of the causal effect of Lp(a) on the outcome.

1. the effect of Lp(a) on sepsis.


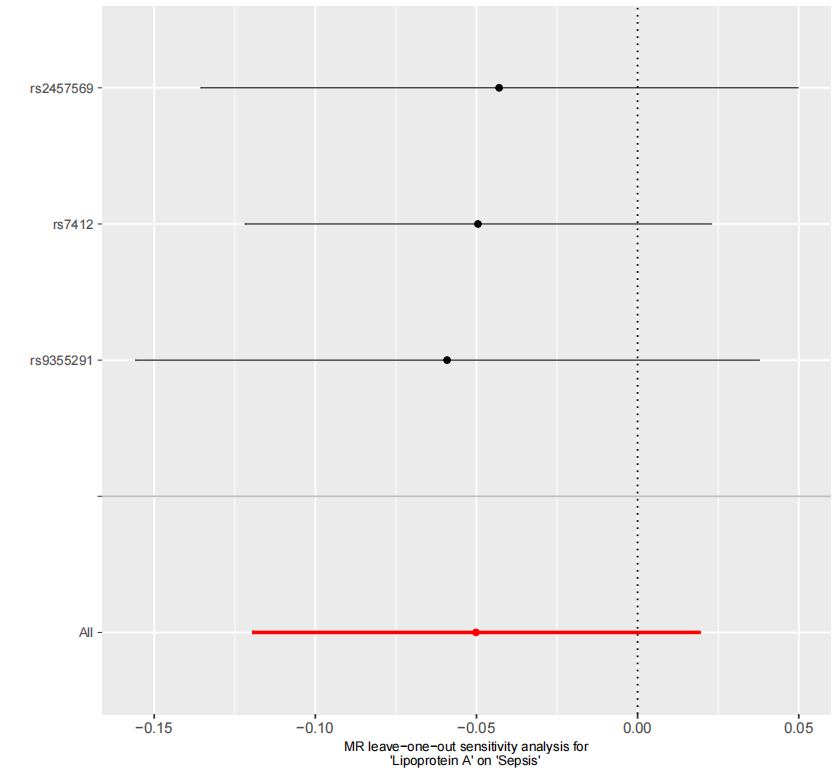


1. the effect of Lp(a) on sepsis (under 75).


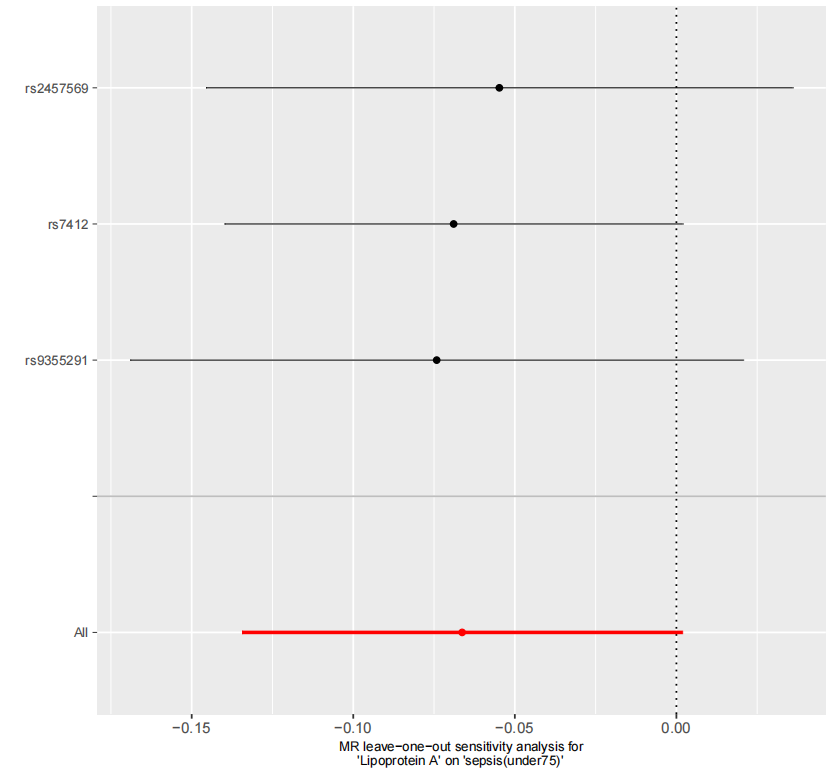


1. the effect of Lp(a) on sepsis (critical care).


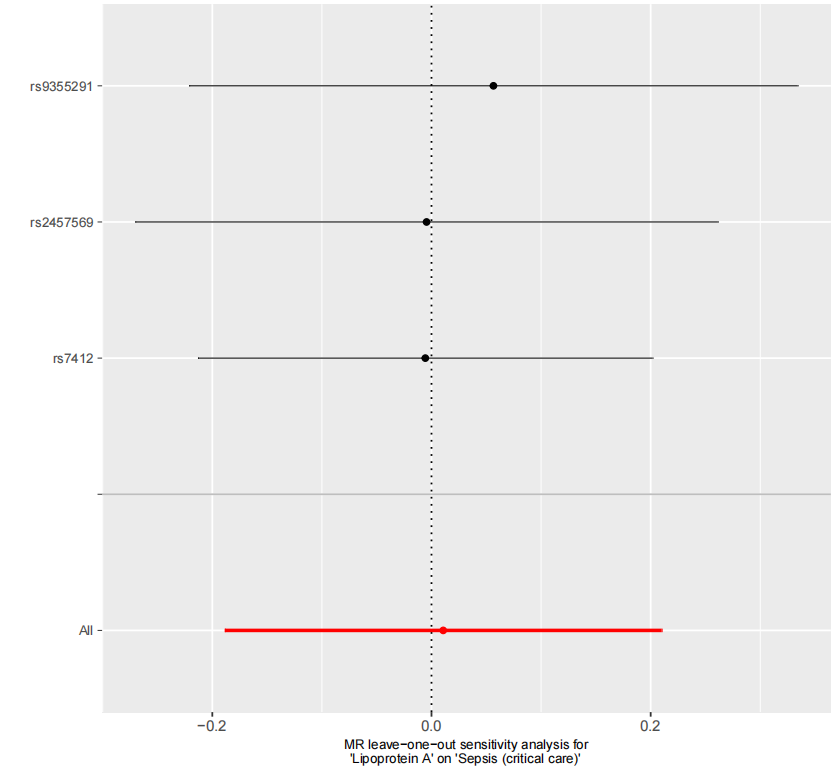


1. the effect of Lp(a) on sepsis (28 day death in critical care).


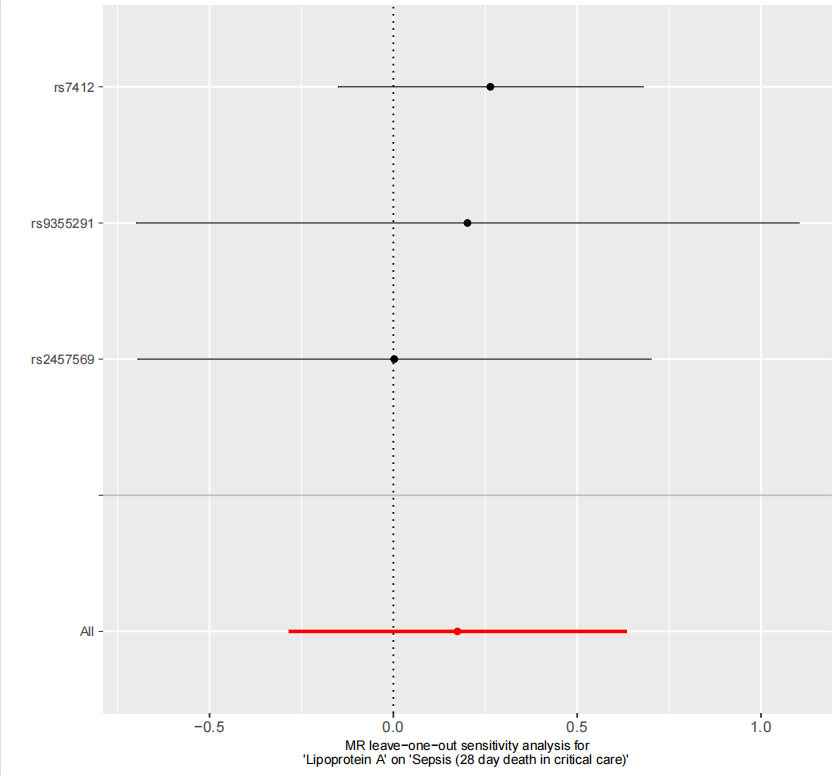

Supplement: S1 File — (DOCX) [file pone.0331023.s001.docx]
